# Use of quantitative molecular diagnostic methods to investigate the effect of enteropathogen infections on linear growth in children in low-resource settings: longitudinal analysis of results from the MAL-ED cohort study

**DOI:** 10.1016/S2214-109X(18)30351-6

**Published:** 2018-10-01

**Authors:** Elizabeth T Rogawski, Jie Liu, James A Platts-Mills, Furqan Kabir, Paphavee Lertsethtakarn, Mery Siguas, Shaila S Khan, Ira Praharaj, Arinao Murei, Rosemary Nshama, Buliga Mujaga, Alexandre Havt, Irene A Maciel, Darwin J Operario, Mami Taniuchi, Jean Gratz, Suzanne E Stroup, James H Roberts, Adil Kalam, Fatima Aziz, Shahida Qureshi, M Ohedul Islam, Pimmada Sakpaisal, Sasikorn Silapong, Pablo P Yori, Revathi Rajendiran, Blossom Benny, Monica McGrath, Jessica C Seidman, Dennis Lang, Michael Gottlieb, Richard L Guerrant, Aldo A M Lima, Jose Paulo Leite, Amidou Samie, Pascal O Bessong, Nicola Page, Ladaporn Bodhidatta, Carl Mason, Sanjaya Shrestha, Ireen Kiwelu, Estomih R Mduma, Najeeha T Iqbal, Zulfiqar A Bhutta, Tahmeed Ahmed, Rashidul Haque, Gagandeep Kang, Margaret N Kosek, Eric R Houpt, The MAL-ED Network Investigators, Angel Mendez Acosta, Rosa Rios de Burga, Cesar Banda Chavez, Julian Torres Flores, Maribel Paredes Olotegui, Silvia Rengifo Pinedo, Dixner Rengifo Trigoso, Angel Orbe Vasquez, Imran Ahmed, Didar Alam, Asad Ali, Muneera Rasheed, Sajid Soofi, Ali Turab, Aisha Yousafzai, Anita KM Zaidi, Binob Shrestha, Bishnu Bahadur Rayamajhi, Tor Strand, Geetha Ammu, Sudhir Babji, Anuradha Bose, Ajila T George, Dinesh Hariraju, M. Steffi Jennifer, Sushil John, Shiny Kaki, Priyadarshani Karunakaran, Beena Koshy, Robin P Lazarus, Jayaprakash Muliyil, Preethi Ragasudha, Mohan Venkata Raghava, Sophy Raju, Anup Ramachandran, Rakhi Ramadas, Karthikeyan Ramanujam, Anuradha Rose, Reeba Roshan, Srujan L Sharma, Shanmuga Sundaram, Rahul J Thomas, William K Pan, Ramya Ambikapathi, J Daniel Carreon, Viyada Doan, Christel Hoest, Stacey Knobler, Mark A Miller, Stephanie Psaki, Zeba Rasmussen, Stephanie A Richard, Karen H Tountas, Erling Svensen, Caroline Amour, Eliwaza Bayyo, Regisiana Mvungi, John Pascal, Ladislaus Yarrot, Leah Barrett, Rebecca Dillingham, William A Petri, Rebecca Scharf, AM Shamsir Ahmed, Md Ashraful Alam, Umma Haque, Md Iqbal Hossain, Munirul Islam, Mustafa Mahfuz, Dinesh Mondal, Baitun Nahar, Fahmida Tofail, Ram Krishna Chandyo, Prakash Sunder Shrestha, Rita Shrestha, Manjeswori Ulak, Aubrey Bauck, Robert Black, Laura Caulfield, William Checkley, Gwenyth Lee, Kerry Schulze, Samuel Scott, Laura E Murray-Kolb, A Catharine Ross, Barbara Schaefer, Suzanne Simons, Laura Pendergast, Cláudia B Abreu, Hilda Costa, Alessandra Di Moura, José Quirino Filho, Álvaro M Leite, Noélia L Lima, Ila F Lima, Bruna LL Maciel, Pedro HQS Medeiros, Milena Moraes, Francisco S Mota, Reinaldo B Oriá, Josiane Quetz, Alberto M Soares, Rosa MS Mota, Crystal L Patil, Cloupas Mahopo, Angelina Maphula, Emanuel Nyathi

**Affiliations:** a Division of Infectious Diseases and International Health, University of Virginia, Charlottesville, VA, USA; b Department of Public Health Sciences, University of Virginia, Charlottesville, VA, USA; c Aga Khan University, Karachi, Pakistan; d Armed Forces Research Institute of Medical Sciences (AFRIMS), Bangkok, Thailand; e Asociación Benéfica PRISMA, Iquitos, Peru; f International Centre for Diarrhoeal Disease Research, Dhaka, Bangladesh; g Christian Medical College, Vellore, India; h University of Venda, Thohoyandou, South Africa; i Haydom Global Health Institute, Haydom, Tanzania; j Kilimanjaro Clinical Research Institute, Moshi, Tanzania; k Federal University of Ceara, Fortaleza, Brazil; l Fundação Oswaldo Cruz (Fiocruz), Rio de Janeiro, Brazil; m Bloomberg School of Public Health, Johns Hopkins University, Baltimore, MD, USA; n Fogarty International Center, National Institutes of Health, Bethesda, MD, USA; o Foundation for the National Institutes of Health, Bethesda, MD, USA; p National Institute for Communicable Diseases, Johannesburg, South Africa; q Walter Reed/AFRIMS Research Unit, Nepal, Kathmandu, Nepal; r University of Bergen, Bergen, Norway

## Abstract

**Background:**

Enteropathogen infections in early childhood not only cause diarrhoea but contribute to poor growth. We used molecular diagnostics to assess whether particular enteropathogens were associated with linear growth across seven low-resource settings.

**Methods:**

We used quantitative PCR to detect 29 enteropathogens in diarrhoeal and non-diarrhoeal stools collected from children in the first 2 years of life obtained during the Etiology, Risk Factors, and Interactions of Enteric Infections and Malnutrition and the Consequences for Child Health and Development (MAL-ED) multisite cohort study. Length was measured monthly. We estimated associations between aetiology-specific diarrhoea and subclinical enteropathogen infection and quantity and attained length in 3 month intervals, at age 2 and 5 years, and used a longitudinal model to account for temporality and time-dependent confounding.

**Findings:**

Among 1469 children who completed 2 year follow-up, 35 622 stool samples were tested and yielded valid results. Diarrhoeal episodes attributed to bacteria and parasites, but not viruses, were associated with small decreases in length after 3 months and at age 2 years. Substantial decrements in length at 2 years were associated with subclinical, non-diarrhoeal, infection with *Shigella* (length-for-age *Z* score [LAZ] reduction −0·14, 95% CI −0·27 to −0·01), enteroaggregative *Escherichia coli* (−0·21, −0·37 to −0·05), *Campylobacter* (−0·17, −0·32 to −0·01), and *Giardia* (−0·17, −0·30 to −0·05). Norovirus, *Cryptosporidium*, typical enteropathogenic *E coli*, and *Enterocytozoon bieneusi* were also associated with small decrements in LAZ. *Shigella* and *E bieneusi* were associated with the largest decreases in LAZ per log increase in quantity per g of stool (−0·13 LAZ, 95% CI −0·22 to −0·03 for *Shigella*; −0·14, −0·26 to −0·02 for *E bieneusi*). Based on these models, interventions that successfully decrease exposure to *Shigella*, enteroaggregative *E coli, Campylobacter*, and *Giardia* could increase mean length of children by 0·12–0·37 LAZ (0·4–1·2 cm) at the MAL-ED sites.

**Interpretation:**

Subclinical infection and quantity of pathogens, particularly *Shigella*, enteroaggregative *E coli, Campylobacter*, and *Giardia*, had a substantial negative association with linear growth, which was sustained during the first 2 years of life, and in some cases, to 5 years. Successfully reducing exposure to certain pathogens might reduce global stunting.

**Funding:**

Bill & Melinda Gates Foundation.

## Introduction

Poor linear growth is an intractable public health issue. Stunting, which is defined as more than two standard deviations below the WHO height-for-age standard,^1^ affects more than 30% of children in low-resource settings.^2^ Impaired linear growth is a cumulative marker of chronic undernutrition that is associated with high mortality,^3^ chronic disease, poor school performance, and low adult economic productivity.^4^, ^5^

Stunting occurs in the context of poverty and results from the interaction of multiple inter-related factors in the prenatal and postnatal environments. Childhood diarrhoea has been recognised as an important determinant of linear growth for decades.^6^, ^7^, ^8^ Diarrhoea due to particular pathogens, such as *Shigella* and enterotoxigenic *Escherichia coli*, has been associated with poor height attainment and poor weight gain, respectively, whereas rotavirus has been shown to have little effect on ponderal or linear growth.^6^, ^9^

**Research in context**

**Evidence before this study**

We searched PubMed for articles published in any language since Jan 1, 1990, using the search terms “(enteric infection) OR enteropathogen OR diarrhea OR diarrhoea)” AND “(pediatric OR paediatric OR infant* OR children)” AND “(grow* OR linear OR height OR length OR stunting OR stunted)” AND “cohort”. With the exception of the Etiology, Risk Factors, and Interactions of Enteric Infections and Malnutrition and the Consequences for Child Health and Development study reported here, we identified 759 publications, of which 20 investigated the association between enteropathogens and child growth. All studies were done at a single site: six focused on aetiology-specific diarrhoea without assessment of subclinical infection and 18 assessed only one pathogen. Only one study used quantitative molecular diagnostic methods to detect enteropathogens.

**Added value of this study**

This study provides a comprehensive, multisite assessment of the effect of enteropathogen infections on linear growth during the first 2 years of life using quantitative PCR for a broad range of enteropathogens in the context of home-based surveillance. These high-resolution data allowed for a rigorous longitudinal analysis that accounted for temporality, lag periods for pathogen effects, and potential confounding by infections with other pathogens.

**Implications of all the available evidence**

Our results showed that subclinical infection and quantity of *Shigella*, enteroaggregative *Escherichia coli, Campylobacter,* and *Giardia* were negatively associated with linear growth, and had larger effect on linear growth than diarrhoeal episodes. Successfully reducing exposure to these pathogens might reduce global stunting.

The Etiology, Risk Factors, and Interactions of Enteric Infections and Malnutrition and the Consequences for Child Health and Development (MAL-ED) study^10^ was a multisite longitudinal birth cohort that assessed the effect of enteric infections and other risk factors on linear growth. In contrast to earlier studies,^6^, ^7^, ^8^ diarrhoea did not have a consistently significant effect on height attainment.^11^, ^12^ Potential reasons included declining diarrhoeal incidence, mild diarrhoeal episodes captured by active surveillance, and high rates of health-care referral. Specific diarrhoeal aetiologies might have had an effect; however, this has not yet been investigated because assigning the aetiology of diarrhoea to specific pathogens is most accurate with the use of quantitative molecular diagnostic testing.^13^

Conversely, subclinical infection with enteropathogens, independent of diarrhoea, was strongly associated with poor height attainment in the MAL-ED study.^11^, ^12^ High prevalence of *Campylobacter* and enteroaggregative *E coli*, the two most prevalent enteropathogens among children in the MAL-ED study, were each associated with approximately 0·8 cm shorter length at 2 years compared with a low prevalence of these pathogens. However, these findings were derived from a subset of stool specimens tested with culture and immunoassay diagnostics, which have lower sensitivity than PCR,^13^ thus pathogen prevalence was underestimated.

We used quantitative molecular diagnostics to assess the association between 29 pathogens and linear growth using stool specimens from the MAL-ED study.

## Methods

### Study design and participants

The MAL-ED study design has been described previously.^10^ Children were enrolled within 17 days of birth at eight locations between November, 2009, and February, 2012. Linear anthropometric measurements were available from seven locations: Dhaka, Bangladesh; Vellore, India; Bhaktapur, Nepal; Fortaleza, Brazil; Loreto, Peru; Venda, South Africa; and Haydom, Tanzania. Children were included if their mother was aged 16 years or older, their family intended to remain in the study area for at least 6 months from enrolment, they were from a singleton pregnancy, they had no other siblings enrolled in the study, and had a birthweight or enrolment weight of more than 1500 g. Children diagnosed with congenital disease or severe neonatal disease were excluded. All sites received ethical approval from their respective governmental, local institutional, and collaborating institutional ethics review boards. Written informed consent was obtained from the parent or guardian of every child.

### Samples

Fieldworkers visited children twice weekly until age 2 years for active surveillance of child illnesses, antibiotic use, breastfeeding, and food intake. Sociodemographic information was collected every 6 months. Linear anthropometric measurements were obtained by fieldworkers monthly to age 2 years (length) and once at age 5 years (±6 months; height).^11^ Diarrhoeal stools were defined by maternal report of three or more loose stools in 24 h or one stool with visible blood. Non-diarrhoeal stool samples were collected monthly (at least 3 days before or after a diarrhoea episode) from birth to age 2 years.

### Stool testing

We tested all stool specimens using custom-designed TaqMan Array Cards (ThermoFisher, Carlsbad, CA, USA) that compartmentalised probe-based quantitative PCR assays for 29 enteropathogens (appendix). Assay validation, nucleic acid extraction, quantitative PCR conditions, and quality control have been previously described.^13^, ^14^ Both *Shigella* and enteroinvasive *E coli* are detected using the *ipaH* target; however, on the basis of previous findings that *Shigella flexneri* and *Shigella sonnei* account for the majority of *ipaH* detections,^13^ and *Shigella* positive stool cultures are metagenomically similar to *ipaH* positive stools,^15^ for simplicity the presence of *ipaH* was considered diagnostic of *Shigella*.

### Procedures

Pathogen-specific aetiology of diarrhoea was determined using attributable fractions (AFe) to adjust for subclinical pathogen infections, as previously described.^13^, ^16^, ^17^ We defined pathogen-attributable episodes when the pathogen quantity-derived AFe was 0·5 or higher (ie, majority attribution). Episodes with a sum of all pathogen-specific AFes of less than 0·5 (ie, the majority of the episode was not attributed to pathogens) were considered non-attributable. We assessed the associations between diarrhoeal aetiologies and growth for diarrhoea episodes attributable to any infection, and to viral, parasitic, and bacterial pathogen groups (appendix). We also assessed individual pathogens, specifically the ten enteropathogens with the highest attributable diarrhoeal incidence in the MAL-ED study (identified in the companion Article^16^): *Shigella*, typical enteropathogenic *E coli, Campylobacter jejuni or Campylobacter coli*, enterotoxigenic *E coli, Cryptosporidium*, astrovirus, sapovirus, norovirus, rotavirus, and adenovirus 40/41. For the subclinical infection and growth analysis, we assessed all 29 pathogens (appendix) and included the 13 most prevalent pathogens as covariates in the models (including all pathogens with significant associations with growth in the height attainment model): enteroaggregative *E coli*, enterotoxigenic *E coli, Giardia, Campylobacter*, atypical enteropathogenic *E coli*, adenovirus 40/41, sapovirus, typical enteropathogenic *E coli*, norovirus, *Shigella*, astrovirus, *Enterocytozoon bieneusi* and *Cryptosporidium*. Length measurements were converted into length-for-age *Z* scores (LAZ) using 2006 WHO child growth standards.^18^ Socioeconomic status was summarised using a construct of water, assets, maternal education, and income^11^, ^15^ and was averaged over four biannual surveys. Exclusive breastfeeding was defined as the proportion of days in a specified time period in which children were breastfed and received no liquids or solids. Potential confounders were included on the basis of previous associations with enteropathogen exposure^19^, ^20^, ^21^ and linear growth.^11^

### Statistical analysis

To estimate the associations between aetiology-specific diarrhoea and linear growth after 3 months, we used repeated measures linear regression with general estimating equations to account for correlation between children's outcomes over time. Models were adjusted for age, site, sex, socioeconomic status, maternal height, LAZ at the beginning of the interval, exclusive breastfeeding, and number of non-attributable diarrhoea episodes in the same period. We also estimated the associations of diarrhoea with fever, dehydration, vomiting, blood, prolonged duration (diarrhoea for 7 days or longer), and high severity (modified Vesikari score >6)^22^ with LAZ after 3 months. We used linear regression to estimate associations between aetiology-specific diarrhoea episodes and LAZ measured at 2 years (acceptable window for measurement was age 731 days ±15 days) in a height attainment model. Models were adjusted for enrolment LAZ, sex, socioeconomic status, exclusive breastfeeding in the first 6 months, maternal height, number of non-attributable diarrhoea episodes, and number of episodes treated with any antibiotics. Effects were estimated for the difference in LAZ at 2 years and scaled to compare a high burden of attributable diarrhoea episodes with a low burden (ie, the difference between the 90th and 10th percentile).

We used linear regression to estimate associations between subclinical enteropathogen infections and LAZ measured at 2 years in a height attainment model, adjusting for site, enrolment LAZ, sex, socioeconomic status, exclusive breastfeeding in the first 6 months, and maternal height. Exposure to each enteropathogen was summarised as the proportion of non-diarrhoeal stools obtained between age 1 and 24 months that were positive for that enteropathogen. The summative effect of pathogen groups was assessed by calculating the mean number of detections between age 1 and 24 months. The difference in LAZ at 2 years associated with each pathogen was scaled to compare the 90th percentile with the 10th percentile for stool positivity (appendix). In a second analysis, we specified pathogen quantity in non-diarrhoeal stools as the exposure, defined by mean log-copy number per g of stool (appendix) and scaled effects per one log increase in pathogen quantity. In a sensitivity analysis, we specified enteropathogen exposure as the proportion of all positive stools (non-diarrhoeal and diarrhoeal) obtained between age 1 and 24 months. We also estimated the associations of enteropathogen exposures with weight-for-age and weight-for-length *Z* scores in models with the same structure, additionally adjusting for enrolment weight-for-age *Z* scores. We estimated the associations with height-for-age *Z* score at 5 years of age (±6 months) in a model with the same structure.

To ensure temporality and include potential lag periods between exposures and growth, we investigated enteropathogens in longitudinal models. We defined exposures in 6 month intervals from birth to 2 years and used the parametric g-formula^23^ to model interim effects on LAZ at the end of the intervals and overall effect on LAZ at 2 years. The parametric g-formula was fitted for each pathogen individually, specified first as the proportion of positive non-diarrhoeal stools and second as the mean quantity in non-diarrhoeal stools during the 6 month interval. Pathogen exposures were assessed with a flexible lag structure including the exposure in the current and previous intervals (appendix). All models were adjusted for the five pathogens with the strongest associations with LAZ at 2 years. We first used the observed data to estimate β-coefficients in longitudinal repeated measures models for each time-dependent covariate in the 6 month intervals (appendix). We used Monte Carlo simulations with the estimated coefficients to predict the time-dependent covariates, pathogen exposures, and LAZ outcomes for each interval to age 2 years in a random sample of 50 000 replicates from the study population at baseline. Simulations were run for high (90th percentile in each interval) and low (10th percentile in each interval) pathogen exposure conditions and the difference due to a one log increase in pathogen quantity. We estimated the cumulative effect of pathogens on LAZ by calculating the difference of the mean predicted outcomes at 2 years between the high and low exposure conditions (population-standardised LAZ difference). 95% CIs were constructed by bootstrapping at the individual level to account for correlation between observations over time with 1000 replicates.

### Role of the funding source

The funder of the study had no role in study design, data collection, data analysis, data interpretation, or writing of the report. The corresponding author had full access to all data in the study and had final responsibility for the decision to submit for publication.

## Results

Among 1868 children enrolled at seven sites, 1469 (79%) were followed up with anthropometry until age 2 years, of whom 1202 had available anthropometry data at age 5 years (±6 months). Mean LAZ within 17 days of birth was slightly less than one standard deviation below the WHO standard, and by age 2 years was almost two standard deviations below the WHO standard (table). We obtained 37 433 stool specimens, of which 35 622 (95%; 30 647 non-diarrhoeal stools and 4975 diarrhoeal stools collected from 94% of diarrhoeal episodes) yielded valid quantitative PCR results for at least one enteropathogen and were included in the analysis.

**Table:** Baseline characteristics, pathogen prevalence, and linear growth outcomes among 1469 children in the MAL-ED cohort with molecular testing of stool samples

		**Dhaka, Bangladesh (n=210)**	**Vellore, India (n=227)**	**Bhaktapur, Nepal (n=227)**	**Venda, South Africa (n=237)**	**Haydom, Tanzania (n=209)**	**Fortaleza, Brazil (n=165)**	**Loreto, Peru (n=194)**	**Total (n=1469)**
Diarrhoeal stools with valid results, n		1390	666	911	132	164	97	1615	4975
	Median per child (IQR)	6 (4–9)	2 (1–4)	3 (2–6)	0 (0–1)	0 (0–1)	0 (0–1)	7 (4–11)	2 (0–5)
Non-diarrhoeal stools with valid results, n		4342	4881	5061	4877	4331	2926	4229	30 647
	Median per child (IQR)	21 (19–22)	22 (21–23)	23 (22–23)	21 (19–22)	21 (20–22)	18 (15–20)	22 (21–23)	21 (20–23)
Sex									
	Female, n (%)	102 (49%)	122 (54%)	105 (46%)	117 (49%)	104 (50%)	76 (46%)	89 (46%)	715 (49%)
	Male, n (%)	108 (51%)	105 (46%)	122 (54%)	120 (51%)	105 (50%)	89 (54%)	105 (54%)	754 (51%)
Improved sanitation, n (%)*		210 (100%)	132 (58%)	227 (100%)	232 (98%)	19 (9%)	165 (100%)	100 (52%)	1084 (74%)
Improved drinking water, n (%)*		210 (100%)	227 (100%)	227 (100%)	216 (91%)	128 (61%)	165 (100%)	192 (99%)	1365 (93%)
Mean maternal height, cm (SD)		145 (5·0)	151 (5·2)	149 (5·2)	158 (6·5)	156 (5·8)	155 (6·7)	150 (5·6)	153 (6·7)
Mean maternal age, years (SD)		25·0 (5·0)	23·9 (4·2)	26·6 (3·7)	27·0 (7·2)	29·1 (6·5)	25·4 (5·6)	24·8 (6·3)	26·0 (5·8)
Mean monthly income <150 US$ (IQR)		132 (62·9)	209 (92·1)	111 (48·9)	44 (18·6)	206 (98·6)	3 (1·8)	138 (71·3)	843 (57·4)
Median months of exclusive breastfeeding (IQR)		5·1 (3·8–5·8)	3·5 (2·4–4·6)	3·0 (1·4–4·4)	1·0 (0·6–1·7)	1·9 (1·2–2·7)	2·6 (1·6–4·4)	2·9 (1·1–4·8)	2·6 (1·3–4·4)
Median number of diarrhoea episodes (IQR)		7 (4–9)	4 (2–6)	4 (2–7)	1 (0–2)	2 (1–4)	1 (0–2)	7·5 (5–12)	3 (1–6)
Mean enrolment WAZ†		−1·26 (0·94)	−1·30 (1·04)	−0·92 (0·97)	−0·38 (0·95)	−0·13 (0·94)	−0·16 (1·05)	−0·62 (0·91)	−0·70 (1·07)
Mean enrolment LAZ†		−0·97 (1·01)	−1·02 (1·05)	−0·72 (1·03)	−0·71 (1·00)	−1·03 (1·14)	−0·80 (1·13)	−0·95 (0·96)	−0·88 (1·05)
Mean WAZ at 2 years		−1·61 (0·99)	−1·65 (0·94)	−0·93 (0·90)	−0·51 (0·98)	−1·33 (1·01)	0·39 (1·21)	−0·79 (0·90)	−0·96 (1·17)
Mean LAZ at 2 years		−2·03 (0·94)	−1·92 (0·97)	−1·35 (0·92)	−1·70 (1·06)	−2·67 (1·02)	−0·04 (1·08)	−1·88 (0·87)	−1·70 (1·20)

MAL-ED=The Etiology, Risk Factors, and Interactions of Enteric Infections and Malnutrition and the Consequences for Child Health and Development study. WAZ=weight-for-age *Z* score. LAZ=length-for-age *Z* scores.

* Individuals with access to improved sanitation and drinking water as defined by WHO guidelines.^24^

† Measured within 17 days of birth.

**Figure 1:**
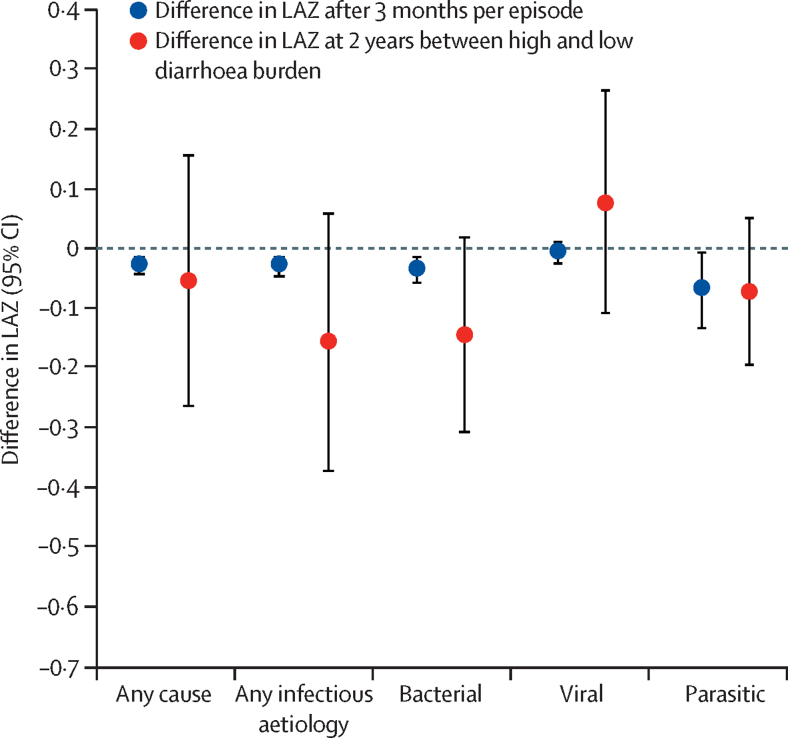
Associations between diarrhoea and LAZ Analysis includes 37 951 observed child-months among 1469 children in the Etiology, Risk Factors, and Interactions of Enteric Infections and Malnutrition and the Consequences for Child Health and Development cohort study who had molecular testing of stool samples. Per-episode estimates were adjusted for age, site, sex, socioeconomic status, maternal height, LAZ at the beginning of the interval, exclusive breastfeeding, and non-attributable diarrhoea episodes in the same period. 2 year estimates were adjusted for site, sex, socioeconomic status, maternal height, enrolment LAZ, exclusive breastfeeding in the first 6 months, number of antibiotic courses, and number of non-attributable diarrhoea episodes. LAZ=length-for-age *Z* scores.

Diarrhoea of any cause occurred in 6296 child-months (mean 4·3 episodes per child between 0 and 2 years), diarrhoea due to any infectious aetiology in 3018 child-months (any quantitative aetiologic pathogen detection; mean 2·1 episodes per child between age 0 and 2 years), bacterial diarrhoea (attributable to enteroaggregative *Escherichia coli*, atypical enteropathogenic *E coli*, typical enteropathogenic *E coli*, enterotoxigenic *E coli*, Shiga toxin-producing *E coli, Aeromonas, C jejuni* or *C coli, Helicobacter pylori, Plesiomonas shigelloides, Salmonella, Shigella*, or *Vibrio cholera*) in 1245 child-months (mean 0·9 episodes per child between age 0 and 2 years), viral diarrhoea (attributable to adenovirus 40/41, astrovirus, norovirus GI or GII, rotavirus, or sapovirus) in 1962 child-months (mean 1·4 episodes per child between age 0 and 2 years), and parasitic diarrhoea (attributable to *E bieneusi, Encephalitozoon intestinalis, Cryptosporidium, Cyclospora, Isospora, Entamoeba histolytica, Giardia, Ancylostoma, Strongyloides*, or *Trichuris*) in 149 child-months (mean 0·1 episodes per child between age 0 and 2 years). Diarrhoea of any cause and infectious diarrhoea episodes were associated with small reductions in LAZ after 3 months (figure 1). The difference in LAZ score varied on the basis of site (appendix) and diarrhoeal aetiology (p_heterogeneity_<0·001). Associations between aetiology-specific diarrhoea and LAZ after 3 months were stronger for bacterial and parasitic diarrhoea than viral diarrhoea. Aetiology-specific episodes attributable to *Shigella* (LAZ difference −0·03, 95% CI −0·05 to −0·00), enterotoxigenic *E coli* (−0·04, −0·07 to −0·01), *Cryptosporidium* (−0·06,–0·13 to 0·00), norovirus (−0·04,–0·08 to 0·00), and adenovirus 40/41 (−0·05, −0·08 to −0·02) were negatively associated with LAZ after 3 months. No short-term associations were identified between LAZ and other specific pathogens (appendix). Diarrhoea episodes with blood (LAZ difference −0·06, 95% CI −0·10 to −0·01), prolonged duration (−0·04, −0·07 to −0·01), and high severity (−0·04, −0·08 to −0·01) were independently associated with reductions in short-term linear growth regardless of aetiology. The cumulative effect of infectious diarrhoea episodes on LAZ at age 2 years was less precise, but showed that a high burden of diarrhoea due to any aetiology was associated with a decrease of −0·16 in LAZ (95% CI −0·37 to 0·06) at age 2 years. Similar to the short-term models, the associations with LAZ varied according to diarrhoeal aetiology (figure 1).

**Figure 2:**
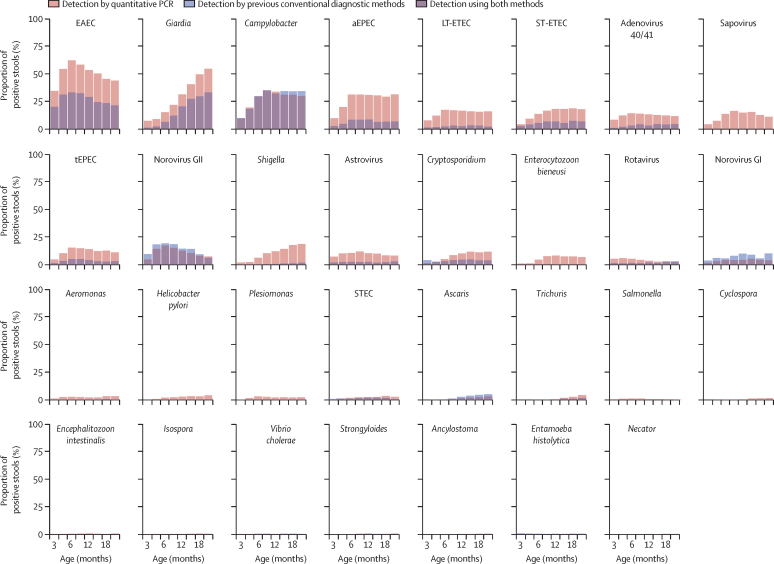
Enteropathogen prevalence in non-diarrhoeal stools obtained from 1469 children in the MAL-ED cohort with molecular testing of stool samples MAL-ED=Etiology, Risk Factors, and Interactions of Enteric Infections and Malnutrition and the Consequences for Child Health and Development. EAEC=enteroaggregative *Escherichia coli*. aEPEC=atypical enteropathogenic *E coli*. LT-ETEC=heat-labile enterotoxigenic *E coli*. ST-ETEC=heat-stable enterotoxigenic *E coli*. tEPEC=typical enteropathogenic *E coli*. STEC=Shiga toxin-producing *E coli*.

**Figure 3:**
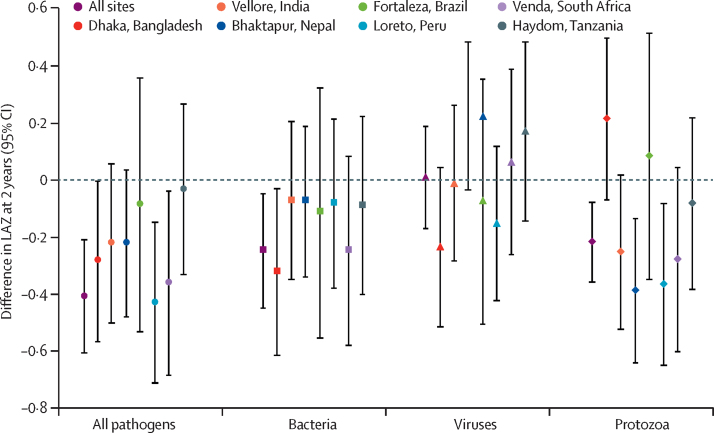
Site-specific effects of enteropathogen infections on height at age 2 years Difference in LAZ at age 2 years between site-specific high and low combined pathogen prevalence in non-diarrhoeal stools among 1469 children in the Etiology, Risk Factors, and Interactions of Enteric Infections and Malnutrition and the Consequences for Child Health and Development cohort. Bacteria include *Campylobacter, Shigella*, enteroaggregative *Escherichia coli*, typical enteropathogenic *E coli*, atypical enteropathogenic *E coli*, and enterotoxigenic *E coli*; viruses include norovirus, adenovirus 40/41, astrovirus, and sapovirus; and protozoa include *Giardia, Cryptosporidium*, and *Enterocytozoon bieneusi* (the latter is an intracellular parasitic fungus). Estimates were adjusted for site, enrolment LAZ, sex, socioeconomic status, exclusive breastfeeding in the first 6 months, and maternal height. LAZ=length-for-age *Z* scores.

**Figure 4:**
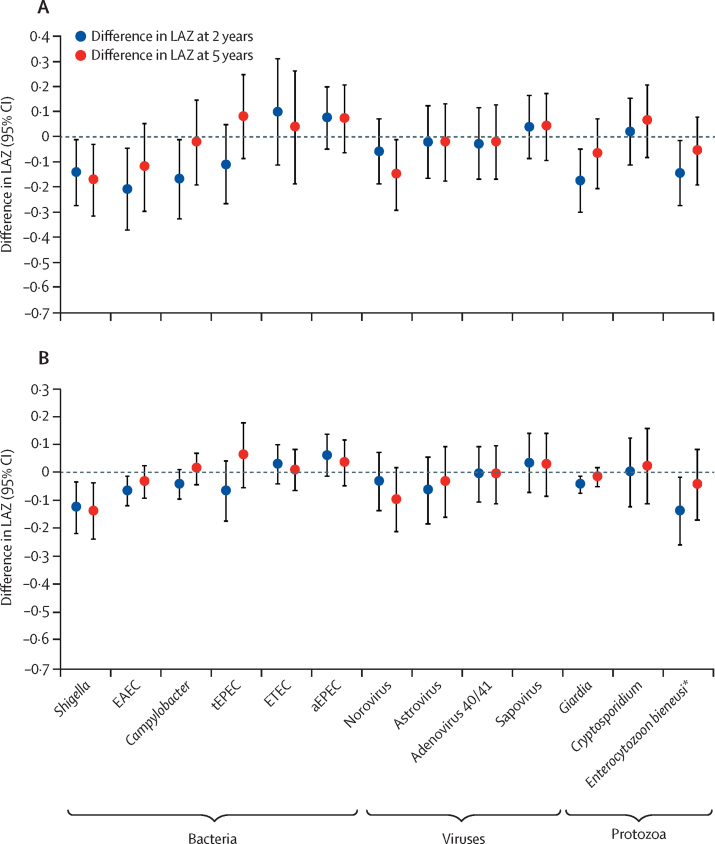
Effect of specific enteropathogen infections on height attainment at age 2 and 5 years Difference in LAZ according to high and low pathogen prevalence (A) and per one log increase in the mean quantity of pathogen per g of stool (B) in non-diarrhoeal stools. Data were available for 1469 children at 2 years and 1202 children at 5 years in the Etiology, Risk Factors, and Interactions of Enteric Infections and Malnutrition and the Consequences for Child Health and Development cohort. Estimates were adjusted for site, enrolment LAZ, sex, SES, exclusive breastfeeding in the first 6 months, and maternal height. LAZ=length-for-age *Z* scores. EAEC=enteroaggregative *Escherichia coli*. tEPEC= typical enteropathogenic *E coli*. ETEC=enterotoxigenic *E coli*. aEPEC=atypical enteropathogenic *E coli*. *Enterocytozoon bieneusi is an intracellular parasitic fungus.

The prevalence of enteropathogen infections detected by quantitative PCR in all non-diarrhoeal stools was higher than that reported previously using previous conventional diagnostic methods, which were mainly culture and immunoassay^17^ (figure 2): of the non-diarrhoeal stools with valid quantitative PCR results, enteroaggregative *E coli* was most prevalent at all sites (15 314 [51%] of 30 034 stools), followed by enterotoxigenic *E coli* (9056 [30%] of 30 172 stools), *Giardia* (8898 [30%] of 30 006 stools) and *Campylobacter* (8398 [28%] of 30 030 stools; appendix). The prevalence of helminths was low (843 [2·8%] of 29 971 stools). Subclinical enteropathogen infections had stronger negative and more significant associations with LAZ at age 2 years than did infectious diarrhoea. A high burden of subclinical infections (90th percentile of detection prevalence) was associated with a LAZ difference of −0·41 (95% CI −0·61 to −0·21) compared with a low burden of subclinical infections (10th percentile; figure 3). Similarly, infections with bacteria and protozoa at the 90th percentile of detection prevalence (combining all sites) were associated with a difference in LAZ of −0·25 (95% CI −0·45 to −0·05) and −0·22 (95% CI −0·36 to −0·08) at 2 years, respectively, compared with detection of these pathogens at the 10th percentile (figure 3). Infections with viruses had no overall effect. High prevalence of enteroaggregative *E coli* (LAZ reduction −0·21, 95% CI −0·37 to −0·05), *Giardia* (−0·17, −0·30 to −0·05), *Campylobacter* (−0·17, −0·32 to −0·01), *Shigella* (−0·14, −0·27 to −0·01), and *E bieneusi* (−0·14, −0·27 to −0·01) in non-diarrhoeal stools were associated with decrements in LAZ at age 2 years (figure 4A). Detection of typical enteropathogenic *E coli* was associated with a small decrement in LAZ, whereas all other pathogens were not associated with LAZ in the height attainment model. The association between enterotoxigenic *E coli* and LAZ did not differ according to the toxins identified, and the association between enteroaggregative *E coli* and LAZ did not change if the detection of the *aggR* gene was used to define enteroaggregative *E coli* instead of *aaiC* or *aatA* genes (data not shown).

*Shigella* and *E bieneusi* were associated with the largest decreases in LAZ (−0·13 LAZ, 95% CI −0·22 to −0·03 for *Shigella*; −0·14,–0·26 to −0·02 for *E bieneusi*) per log increase in quantity per g of stool (figure 4B). Enteroaggregative *E coli, Giardia*, and *Campylobacter* were associated with smaller decrements in LAZ using this exposure specification (ie, quantity rather than prevalence). In the subset of 1202 children with height measurements at age 5 years, most associations between pathogen burden in the first 2 years of life and height-for-age *Z* scores at 5 years were slightly smaller than those at 2 years (figure 4), with the exception of *Shigella* and norovirus, for which the associations were sustained or increased. The inclusion of diarrhoeal stools in the measure of pathogen exposure did not increase the effects on LAZ observed for non-diarrhoeal stools alone (appendix). Associations between pathogens and weight-for-age and weight-for-length *Z* scores were similar (appendix). When comparing the findings derived from the quantitative PCR detection methods versus the conventional diagnostics of culture and immunoassay, the most notable difference was for *Shigella*, which was not associated with LAZ using the conventional diagnostics because of the relative insensitivity of culture (appendix).

**Figure 5:**
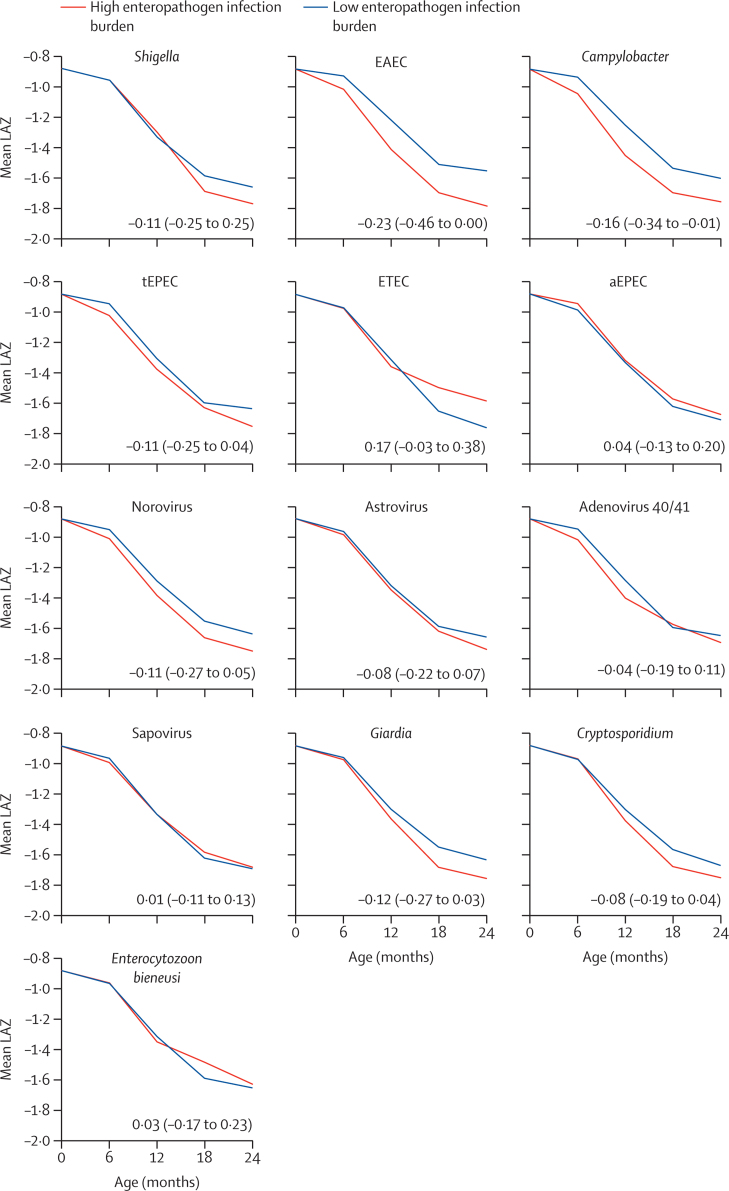
Mean LAZ from birth to age 2 years in the high and low enteropathogen infection burden parametric g-formula conditions The LAZ difference and 95% CI comparing the two conditions at 2 years is overlaid on each graph, for the 13 most prevalent enteropathogens in non-diarrhoeal stools. LAZ=length-for-age *Z* scores. EAEC=enteroaggregative *Escherichia coli*. tEPEC=typical enteropathogenic *E coli*. ETEC=enterotoxigenic *E coli*. aEPEC=atypical enteropathogenic *E coli*.

In the logitudinal model, mean LAZ at each interval for the high and low pathogen exposure conditions showed that the largest differences in length associated with pathogen exposure accrued in the second year of life were coincident with increases in pathogen prevalence (figure 5). These models generally supported the results from the height attainment model. For example, the population-standardised LAZ difference at age 2 years between the high and low enteroaggregative *E coli* exposure conditions was −0·23 (95% CI −0·46 to 0·00), similar to the estimate from the height attainment model. The negative differences in LAZ observed at age 2 years were smaller and 95% CIs were wide for *Shigella, Giardia, Campylobacter*, and typical enteropathogenic *E coli*. High prevalence of *Cryptosporidium*, norovirus, and astrovirus, which were not associated with LAZ in the height attainment model, were associated with small decrements in LAZ by age 2 years in the longitudinal model. Although *E bieneusi* had a significant association in the height attainment model (LAZ difference −0·14, 95% CI −0·27 to −0·01), no association was identified in the longitudinal model (LAZ difference at 2 years 0·03, 95% CI −0·17 to −0·23). The longitudinal models showed little or no effect attributable to adenovirus 40/41, sapovirus, atypical enteropathogenic *E coli*, or enterotoxigenic *E coli*. The effects of pathogen quantities on LAZ was similar to that of pathogen prevalence; the per log quantity-based effect was slightly larger for *Shigella* and astrovirus, and smaller for *Campylobacter,* enteroaggregative *E coli*, and *Giardia* (appendix).

An intervention that could prevent all exposure to the four pathogens most consistently associated with poor linear growth (*Shigella*, enteroaggregative *E coli, Campylobacter*, and *Giardia*), would be expected to increase mean LAZ at 2 years by 0·24 (95% CI 0·08 to 0·41; 0·75 cm). The effect would be expected to range from 0·12–0·37 LAZ (0·4–1·2 cm) across sites, with the largest effect at sites with a high burden of enteropathogens and stunting.

## Discussion

The incorporation of sensitive and quantitative molecular diagnostics to identify both symptomatic and subclinical enteric infections across seven diverse sites allowed a high-resolution examination of the effects of these pathogens on childhood growth. Although diarrhoea episodes, including pathogen-attributable episodes, had small short-term associations with length, their associations with length were smaller at 2 years and were less than half the magnitude of those for subclinical infections. Conversely, subclinical infections of certain enteropathogens were associated with substantial and sustained linear growth deficits.

Subclinical infections with bacteria had larger and more consistent associations with larger decrements in LAZ than those of viruses or protozoa. Not all bacteria had the same effect: enteroaggregative *E coli, Campylobacter*, and *Shigella* infection resulted in the largest population-level differences in LAZ. The associations between decrements in LAZ and enteroaggregative *E coli*^21^ and *Campylobacter*^19^ have been described previously using previous diagnostic testing methods and were confirmed in this study. By contrast, the association between *Shigella* prevalence and decrement in LAZ score was an important finding that has not been identified previously using cell culture,^11^ which might be due to the relative insensitivity of *Shigella* culture. Similarly, we were able to detect *E bieneusi* in this analysis because of the broad PCR-based approach. These findings reflect a combined metric determined by both the pathogen's prevelance at the population-level, regardless of quantity, and its association with poor growth. When pathogen quantity was examined independently of prevalence, *Shigella* had the largest association with decrements in LAZ, suggesting it is the most potently deleterious pathogen for growth. *Shigella* infections in the first 2 years of life also had a sustained effect on height at age 5 years. Our analysis also identified an association between *Giardia* and poor linear growth not apparent previously,^20^ which is likely to be a result of monthly sampling in the second year of life, which was more frequent than in previous analyses.^20^ Helminths have traditionally been associated with malnutrition,^25^ but prevalence was low (2·8%) in this age group, resulting in null and imprecise associations. A previous study^26^ reported that *Cryptosporidium* is associated with poor growth, consistent with our results in the longitudinal model.

Although the mechanisms by which subclinical infections lead to impaired growth are not fully defined, the pathway is thought to involve environmental enteric dysfunction and inflammation.^5^, ^27^ *Shigella* has long been associated with inflammatory diarrhoea. *Campylobacter*^19^ and enteroaggregative *E coli*^21^ were also associated with intestinal inflammation in the MAL-ED study, and *Giardia* was associated with intestinal permeability and malabsorption.^20^ *E bieneusi* has been associated with intestinal malabsorption in patients with HIV.^28^ The association between norovirus infection and short height at 5 years, and low weight-for-age *Z* score and weight-for-height *Z* score at 2 years, differs from the other viruses and merits further investigation.

Based on these observational data, a public health intervention that could prevent infections with *Shigella*, enteroaggregative *E coli, Campylobacter*, and *Giardia* at the MAL-ED sites would be expected to increase mean length of children at 2 years by 0·75 cm, with a larger effect (1·2 cm) observed at sites with high pathogen burden and stunting. The magnitude of this difference is similar to or exceeds that of nutritional supplementation trials (observed increase in LAZ of approximately 0·25),^29^, ^30^, ^31^ and this estimate requires experimental investigation. Water, sanitation, and hygiene interventions seem promising, but large trials^29^, ^30^ have shown little effect on linear growth. It would be useful to assess how well these interventions reduce subclinical enteropathogen infections. Vaccines are not available for any of these four pathogens; however, *Shigella*^32^ and *C jejuni* or *C coli*^33^ vaccines are in early phase development to prevent diarrhoea. How effectively these vaccines will reduce subclinical infection is unclear. Timing could be important, since the longitudinal model suggests that the growth impact of *Shigella* and *Giardia* occurs mostly in the second year of life, whereas *Campylobacter* and enteroaggregative *E coli* have an effect at an earlier age. Antimicrobial strategies could be tested, since evidence^34^, ^35^ indicates that antibiotics can improve child weight and linear growth, with careful consideration of antimicrobial resistance. Considering *Shigella*'s consistent, potent, and sustained associations with low LAZ, we speculate that improved diagnosis and treatment of *Shigella* diarrhoea could be particularly useful to target *Shigella* infection and transmission.^16^

Strengths of this study include the equitable quantitative PCR approach for all 29 enteropathogens, which enabled us to compare and control for other pathogens. The use of multiple statistical models allowed us to assess the consistency of findings. We believe the longitudinal model is the least biased because it reduces the potential for reverse causality (ie, that undernutrition increases risk of infections) by ensuring the directionality from pathogen exposure to growth in 6 month periods. For example, the association of *E bieneusi* in the height attainment but not longitudinal models suggests that rather than the pathogen causing poor linear growth, undernourished children are infected with *E bieneusi*. Consistency between the longitudinal and height attainment models for most other pathogens suggests reverse causality does not explain these associations. The longitudinal model also allows visualisation of catch-up growth, highlighted by adenovirus 40/41. Additionally, the incorporation of multiple time periods for the same child with and without diarrhoea limits confounding by fixed child-specific factors such as socioeconomic status.

A limitation of the longitudinal model is that estimates were less precise than those from the height attainment model, driven by the relatively small number of children sampled at each site. Another limitation of this study is that rare but severe infections, and infections in the first year of life when enteropathogens were less prevalent, would be expected to have little effect at the population level, but could still have substantial effects at the individual level. Additionally, most diarrhoeal episodes captured in this community-based observational cohort were mild; therefore, we might be underestimating the growth impact of diarrhoea in populations with more severe disease.

A previous study^36^ has shown that the morbidity burden of childhood diarrhoea increases substantially when secondary effects on malnutrition are included in estimates. Our results indicate that even these diarrhoea morbidity estimates are substantially underestimating the full impact of enteropathogens by not considering subclinical infections. Moreover, specific enteropathogens have varying effects. Some enteropathogens are associated primarily with poor growth attainment (eg, enteroaggregative *E coli* and *Giardia*), some are associated with both diarrhoea and poor growth (eg, *Shigella, Campylobacter*, and norovirus), and some are associated primarily with diarrhoea (eg, rotavirus, sapovirus, adenovirus, and enterotoxigenic *E coli*).

In summary, focused strategies to prevent or reduce subclinical infection duration or quantity of a small number of select enteropathogens might contribute to reductions in stunting globally. Reducing the full burden of enteropathogens will require novel interventions that can tackle not only diarrhoea but also subclinical infections.
