# Supplementary material for: Use of quantitative molecular diagnostic methods to investigate the effect of enteropathogen infections on linear growth in children in low-resource settings: longitudinal analysis of results from the MAL-ED cohort study
Source: Lancet Glob Health. 2018 Oct 1;6(12):e1319–28. doi: 10.1016/S2214-109X(18)30351-6 (PMC6227248; doi:10.1016/S2214-109X(18)30351-6)
Supplement: Supplementary appendix [file mmc1.pdf]

THE LANCET
Global Health

Supplementary appendix

This appendix formed part of the original submission and has been peer reviewed. We post it as supplied by the authors.

Supplement to: Rogawski ET, Liu J, Platts-Mills JA, et al, and The MAL-ED Network Investigators. Use of quantitative molecular diagnostic methods to investigate the effect of enteropathogen infections on linear growth in children in low-resource settings: longitudinal analysis of results from the MAL-ED cohort study. *Lancet Glob Health* 2018; published online Oct 1. http://dx.doi.org/10.1016/S2214-109X(18)30351-6.

Supplementary Material

Additional MAL-ED Investigators 2

Methods 3

Table S1. Real time PCR assays on TaqMan Array Card used for MAL-ED diarrhoea aetiology and growth analysis. 3

Longitudinal model methods and validation 4

Figure S1. Schematic diagram of the outcome models for the parametric g-formula. 5

Figure S2. Validation of parametric g-formula models for pathogen exposures. 6

Figure S3. Validation of parametric g-formula models for time-varying covariates and LAZ outcomes. 7

Enteropathogen prevalence and quantity 8

Table S2. Enteropathogen prevalence in non-diarrhoeal stools during the first 2 years of life by site among 1469 children in the MAL-ED cohort. 8

Table S3. Enteropathogen quantity in positive non-diarrhoeal stools during the first 2 years of life by site among 1469 children in the MAL-ED cohort. 9

Table S4. Enteropathogen prevalence per child in 6 month intervals during the first 2 years of life among 1469 children in the MAL-ED cohort. 10

Table S5. Enteropathogen quantity per child during the first 2 years of life among 1469 children in the MAL-ED cohort. 11

Figure S4. Associations of pathogen-attributable diarrhoea with length-for-age z-score. 12

Site-specific effects 13

Table S6. Site-specific associations of pathogen-attributable diarrhoea episodes on LAZ 3 months following the episode 13

Table S7. Site-specific differences in length-for-age z-score at 2 years of age associated with the difference between high (site-specific 90th percentile) and low (site-specific 10th percentile) exposure to each of the 13 most prevalent enteropathogens in non-diarrhoeal stools among 1469 children in the MAL-ED cohort. 14

Height attainment model sensitivity analyses 15

Figure S5. Height attainment model: qPCR vs. conventional methods. 15

Figure S6. Height attainment model: non-diarrhoeal stools and diarrhoeal stools. 16

Figure S7. Height attainment model: weight-for-age (WAZ) and weight-for-length (WLZ) z-scores. 17

Longitudinal model sensitivity analyses 18

Figure S8. Longitudinal g-formula: effects scaled per one log increase in pathogen quantity. 18

References 19

Additional MAL-ED Investigators

Angel Mendez Acosta[1]
Rosa Rios de Burga[1]
Cesar Banda Chavez[1]
Julian Torres Flores[1]
Maribel Paredes Olotegui[1]
Silvia Rengifo Pinedo[1]
Dixner Rengifo Trigoso[1]
Angel Orbe Vasquez[1]
Imran Ahmed[2]
Didar Alam[2]
Asad Ali[2]
Muneera Rasheed[2]
Sajid Soofi[2]
Ali Turab[2]
Aisha Yousafzai[2]
Anita KM Zaidi[2]
Binob Shrestha[3]
Bishnu Bahadur Rayamajhi[3]
Tor Strand[3,19]
Geetha Ammu[4]
Sudhir Babji[4]
Anuradha Bose[4]
Ajila T George[4]
Dinesh Hariraju[4]
M. Steffi Jennifer[4]
Sushil John[4]
Shiny Kaki[4]
Priyadarshani Karunakaran[4]
Beena Koshy[4]
Robin P Lazarus[4]
Jayaprakash Muliyil[4]
Preethi Ragasudha[4]
Mohan Venkata Raghava[4]
Sophy Raju[4]
Anup Ramachandran[4]
Rakhi Ramadas[4]
Karthikeyan Ramanujam[4]
Anuradha Rose[4]
Reeba Roshan[4]
Srujan L Sharma[4]
Shanmuga Sundaram E[4]
Rahul J Thomas[4]
William K Pan[5,6]
Ramya Ambikapathi[6,15]
J Daniel Carreon[6]
Viyada Doan[6]
Christel Hoest[6]
Stacey Knobler[6]
Benjamin JJ McCormick[6]
Mark A Miller[6]
Stephanie Psaki[6]
Zeba Rasmussen[6]
Stephanie A Richard[6]
Karen H Tountas[7]
Erling Svensen[8,9]
Caroline Amour[9]
Eliwaza Bayyo[9]
Regisiana Mvungi[9]
John Pascal[9]
Ladislaus Yarrot[9]
Leah Barrett[10]
Rebecca Dillingham[10]
William A Petri, Jr[10]
Rebecca Scharf[10]
AM Shamsir Ahmed[11]
Md Ashraful Alam[11]
Umma Haque[11]
Md Iqbal Hossain[11]
Munirul Islam[11]
Mustafa Mahfuz[11]
Dinesh Mondal[11]
Baitun Nahar[11]
Fahmida Tofail[11]
Ram Krishna Chandyo[12]
Prakash Sunder Shrestha[12]
Rita Shrestha[12]
Manjeswori Ulak[12]
Aubrey Bauck[13]
Robert Black[13]
Laura Caulfield[13]
William Checkley[13,6]
Gwenyth Lee[13,21]
Kerry Schulze[13]
Samuel Scott[14]
Laura E. Murray-Kolb[16]
A Catharine Ross[16]
Barbara Schaefer[16,6]
Suzanne Simons[16]
Laura Pendergast[17]
Cláudia B Abreu[18]
Hilda Costa[18]
Alessandra Di Moura[18]
José Quirino Filho[18,7]
Álvaro M Leite[18]
Noélia L Lima[18]
Ila F Lima[18]
Bruna LL Maciel[18]
Pedro HQS Medeiros[18]
Milena Moraes[18]
Francisco S Mota[18]
Reinaldo B Oriá[18]
Josiane Quetz[18]
Alberto M Soares[18]
Rosa MS Mota[18]
Crystal L Patil[20]
Cloupas Mahopo[22]
Angelina Maphula[22]
Emanuel Nyathi[22]

[1]A.B. PRISMA, Iquitos, Peru
[2]Aga Khan University, Karachi, Pakistan
[3]Walter Reed/AFRIMS Research Unit, Kathmandu, Nepal
[4]Christian Medical College, Vellore, India
[5]Duke University, Durham, NC, USA
[6]Fogarty International Center/National Institutes of Health, Bethesda, MD, USA
[7]Foundation for the National Institutes of Health, Bethesda, MD, USA
[8]Haukeland University Hospital, Bergen, Norway
[9]Haydom Lutheran Hospital, Haydom, Tanzania
[10]University of Virginia, Charlottesville, VA, USA
[11]icddr,b, Dhaka, Bangladesh
[12]Institute of Medicine, Tribhuvan University, Kathmandu, Nepal
[13]Johns Hopkins University, Baltimore, MD, USA
[14]Poverty Health and Nutrition Division, International Food Policy Research Institute, Washington DC, USA
[15]Purdue University, Department of Nutrition Science, IN, USA
[16]The Pennsylvania State University, University Park, PA, USA
[17]Temple University, Philadelphia, PA, USA
[18]Universidade Federal do Ceara, Fortaleza, Brazil
[19]University of Bergen, Norway
[20]University of Illinois at Chicago, IL, USA
[21]University of Michigan, Department of Epidemiology, MI, USA
[22]University of Venda, Thohoyandou, South Africa

Methods

Table S1. Real time PCR assays on TaqMan Array Card used for MAL-ED diarrhoea aetiology and growth analysis.

All the assays have been described previously and extensively validated.[1–3] Nucleic acid was extracted with the QIAamp Fast DNA Stool mini kit (Qiagen, Hilden, Germany) with pre-treatment steps that included bead beating. AgPath One Step RT-PCR reagents were used for qPCR reactions, which were performed on ViiA 7, or QuantStudio 7, or QuantStudio 12K Flex systems. Quantification cycles (Cqs) are the PCR cycle values at which fluorescence from amplification exceeds the background, which acts as an inverse metric of quantity of nucleic acid. Valid results required proper functioning of controls (the negative results of a sample are valid only when its external control MS2 is positive, Cq ≤ 35; the positive results are valid only when the corresponding extraction blank is negative for the relevant targets, Cq > 35), and excluded data flagged by the real time PCR software, i.e. BADROX in combination with NOISE or SPIKE.

	Pathogen	Gene	Included in diarrhoea-growth analysis	Included in infection-growth analysis	Assay in the original microbiologic work-up (conventional methods)*
Viruses	Adenovirus 40/41	Fiber gene	✓	✓	EIA†
	Astrovirus	Capsid	✓	✓	EIA
	Norovirus GI/GII	GI ORF1-2 and GII ORF1-2	✓	✓	PCR§
	Rotavirus	*NSP3*	✓		EIA
	Sapovirus	*RdRp*	✓	✓	Not tested
Bacteria	EAEC**	*aaiC, aatA, aggR*	✓	✓	Culture/PCR
	Atypical EPEC**	*eae*	✓	✓	Culture/PCR
	Typical EPEC**	*bfpA*	✓	✓	Culture/PCR
	ETEC**	*LT*, *STh* and *STp*	✓	✓	Culture/PCR
	STEC**	*stx1, stx2*	✓		Culture/PCR
	Aeromonas	Aerolysin	✓		Culture
	Campylobacter spp.	*cadF (C. jejuni/coli)* and *cpn60* (*Campylobacter* spp)	✓ (*C. jejuni/coli*)	✓ (*Campylobacter* spp)	EIA††
	Helicobacter pylori	*ureC*	✓		Not tested
	Plesiomonas shigelloides	*gyrB*	✓		Culture
	Salmonella	*ttr*	✓		Culture
	Shigella/EIEC	*ipaH*	✓	✓	Culture
	Vibrio cholerae	*hlyA*	✓		Culture
Fungi	*Enterocytozoon bieneusi*	*ITS*	✓		Not tested
	Encephalitozoon intestinalis	SSU rRNA	✓		Not tested
Protozoa	*Cryptosporidium*	18S rRNA	✓	✓	EIA
	Cyclospora cayetanensis	18S rRNA	✓		Microscopy
	Cystoisospora belli	18S rRNA	✓		Microscopy
	Entamoeba histolytica	18S rRNA	✓		EIA
	Giardia	18S rRNA	✓	✓	EIA
Helminth	*Ancylostoma duodenale*	*ITS2*	✓		Microscopy
	Ascaris lumbricoides	*ITS1*			Microscopy
	Necator americanus	*ITS2*			Microscopy
	Strongyloides stercoralis	Dispersed repetitive sequence	✓		Microscopy
	Trichuris trichiura	18S rRNA	✓		Microscopy
Controls	MS2	*MS2g1*			N/A
	PhHV	*gB*			N/A

* Conventional methods were conducted in non-diarrhoeal stools monthly in the first year of life, but only quarterly in the second year of life (qPCR was conducted in non-diarrhoeal stools monthly for both years).

† Pan-adenovirus EIA only.

§ Norovirus PCR was only performed on diarrhoeal and non-diarrhoeal stools from a randomly selected 10% of participants.

***E. coli* pathotypes were defined as follows: EAEC (*aaiC*, or *aatA*, or both), atypical EPEC (*eae* without *bfpA*, *stx1*, and *stx2*), typical EPEC (*bfpA*), ETEC (STh, STp, or LT), STEC (*eae* without *bfpA* and with *stx1*, *stx2*, or both). EAEC was alternatively defined by *aggR* in sensitivity analysis.

††A single EIA was used, which has been shown to detect some *Campylobacter* species other than *C. jejuni* and *C. coli*.[4]

Longitudinal model methods and validation

All analyses were conducted in SAS version 9.4 (Cary, North Carolina, USA). To implement the parametric g-formula, we first used the observed data to estimate β-coefficients in a series of longitudinal repeated measures models for each time-varying covariate in the 6-month intervals. Results from models with 3-month and 6-month intervals were largely consistent; we present the 6-month interval results because 3-month interval-based estimates were less precise and more sensitive to model specification. All models included an indicator for the interval, LAZ at the beginning of the interval, site, sex, and SES as covariates. The time-varying covariates modelled were:

1. Percent days with exclusive breastfeeding: modelled with Poisson regression with an offset for the number of days under surveillance in the period, and also adjusting for maternal age.
2. Incidence of diarrhoea (number of episodes): modelled with Poisson regression and also adjusting for exclusive breastfeeding, and detection of *Campylobacter*, *Shigella*, and tEPEC in the previous interval.
3. Macrolide use (yes/no): modelled with logistic regression and also adjusting for exposure to EAEC, *Campylobacter*, *Giardia*, *Shigella*, and tEPEC in the previous interval, number of diarrhoea episodes, exclusive breastfeeding, and improved sanitation. Macrolide use was included since it was a strong predictor of *Campylobacter*, *Shigella*, and tEPEC infection.
4. The number of detections of pathogens that were not the exposure of interest (*Campylobacter* spp., *Giardia*, EAEC, tEPEC, and *Shigella*): modelled with Poisson regression with an offset for the number of stool samples tested for that pathogen, and also adjusting for exposure to that pathogen in the previous interval, exposure to the other four pathogens in the previous interval, exclusive breastfeeding, number of diarrhoea episodes, macrolide use, maternal height, improved sanitation, and improved drinking water.
5. The average quantity of these pathogens: modelled with the same covariates using log-linear regression only among those with positive detections.
6. The number of detections or average quantity of the pathogen of interest: similarly modelled with Poisson and log-linear regression, respectively, but further adjusted for exposure to the other pathogens (from step 4) in the current interval.
7. The outcome, LAZ, at the end of the interval: modelled using a separate linear regression model for each 6-month time period, and additionally adjusted for the current and lagged pathogen exposure variables (no lags during the first 6-month period, and progressively more lags for later periods), exclusive breastfeeding (first period only), and maternal height (Figure S1).

These time-varying covariates were considered for inclusion based on a causal directed acyclic graph.[4] Covariates were dropped from models for specific pathogen detections if they did not have a strong (|LAZ difference| > 0.02) or significant association ($p < 0.05$) with that pathogen and if model fit improved by Akaike information criterion (AIC).

We then used Monte Carlo simulations with the estimated coefficients from the models above to predict the time-varying covariates, pathogen exposures, and LAZ outcomes for each interval to 2 years of age in a random sample of 50,000 replicates from the study population at baseline. The simulation was completed for the natural course scenario, in which all pathogen exposures were predicted based on the observed data, to validate model fit. The simulated data under the natural course scenario fit the observed data well, such that the interval-specific distributions of the time-varying covariates and LAZ outcomes were similar between the observed and simulated data (Figure S2).

The simulations were then completed for high and low pathogen exposure scenarios, which produced the predicted covariates and outcomes had all children been exposed to high pathogen exposure, defined by the 90th percentile in each interval, versus had all children been exposed to low pathogen exposure, defined by the 10th percentile in each interval. To estimate the cumulative effect of pathogen exposure on LAZ at 2 years of age, we averaged the predicted LAZ outcomes at 2 years across individuals in the high and low pathogen exposure scenarios and took their difference to estimate the population-standardized LAZ difference. A similar process was used to estimate the cumulative effect of pathogen exposure on LAZ at 2 years of age per one log increase in pathogen quantity. Confidence intervals were constructed by bootstrap of the above steps with 1000 replicates.

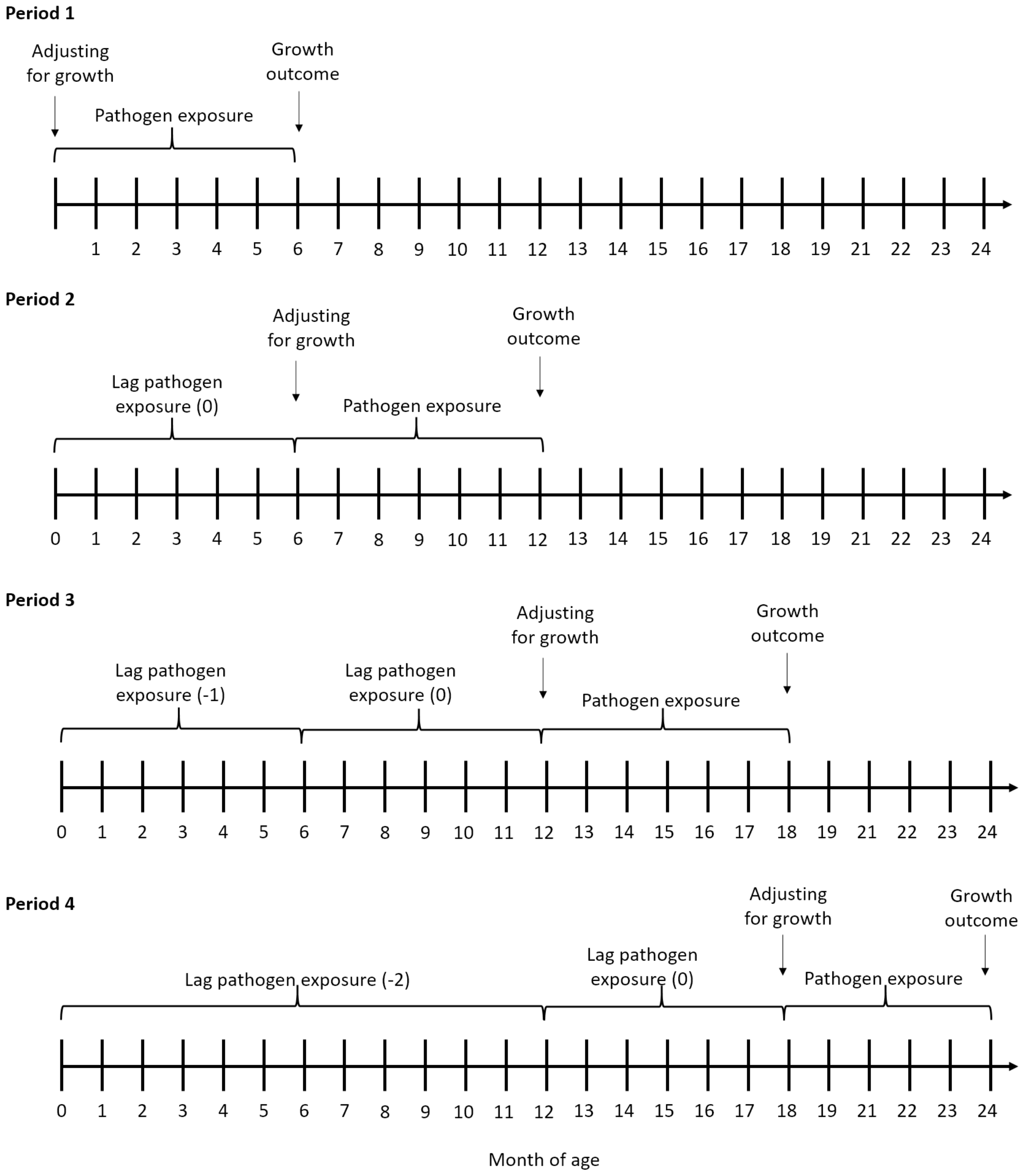

Figure S1. Schematic diagram of the outcome models for the parametric g-formula.

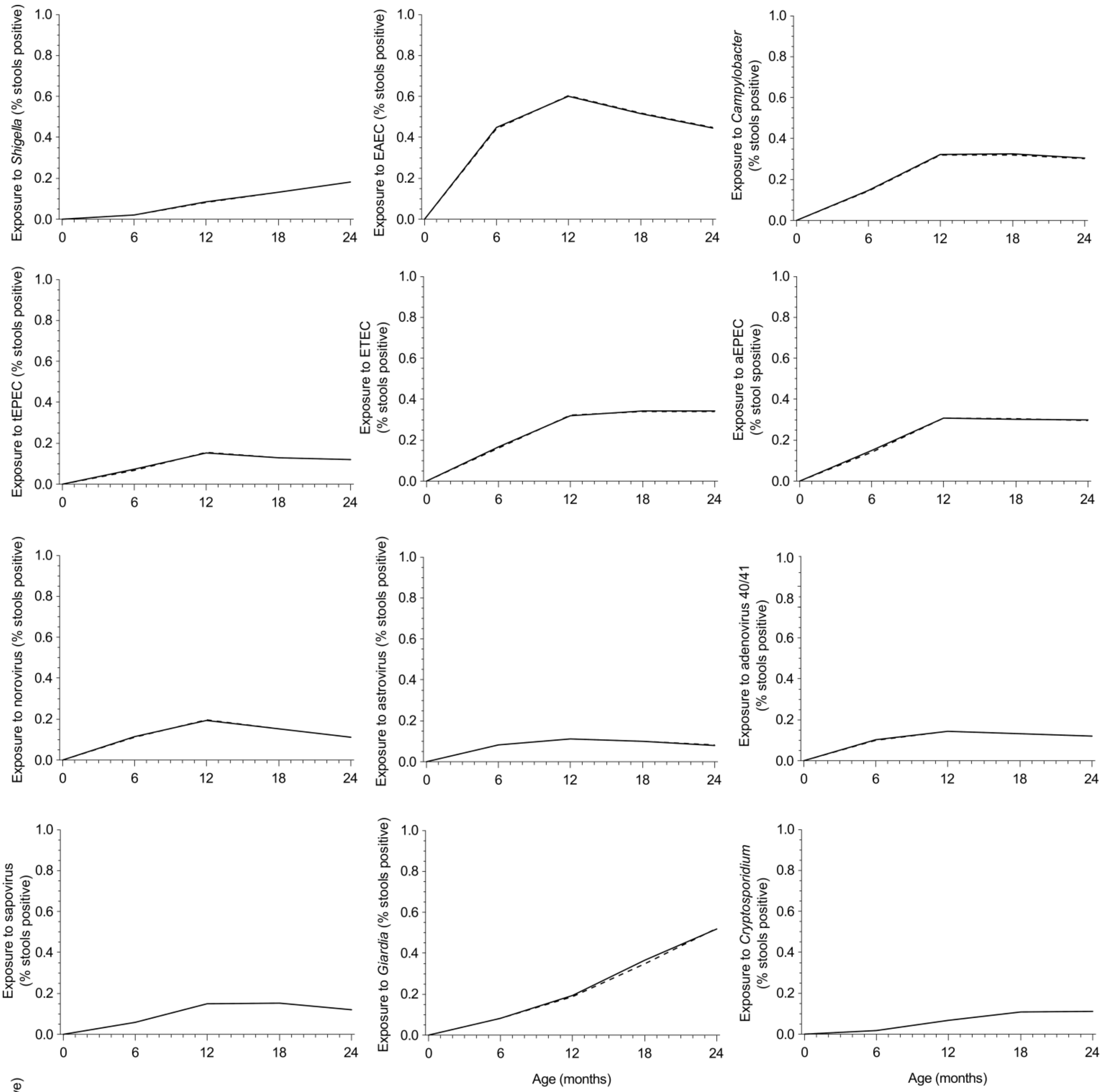

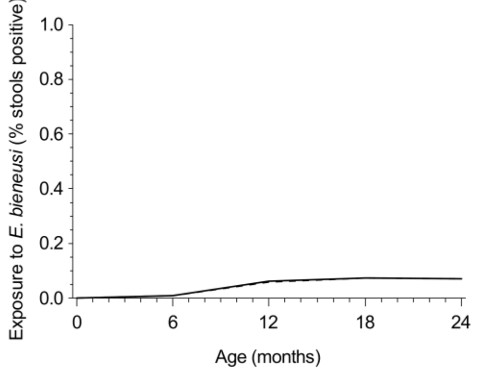

Figure S2. Validation of parametric g-formula models for pathogen exposures.

Average exposure to enteropathogens from birth to 2 years of age in the observed data (solid line) and natural course parametric g-formula scenario (dotted line) based on 1469 children in the MAL-ED cohort.

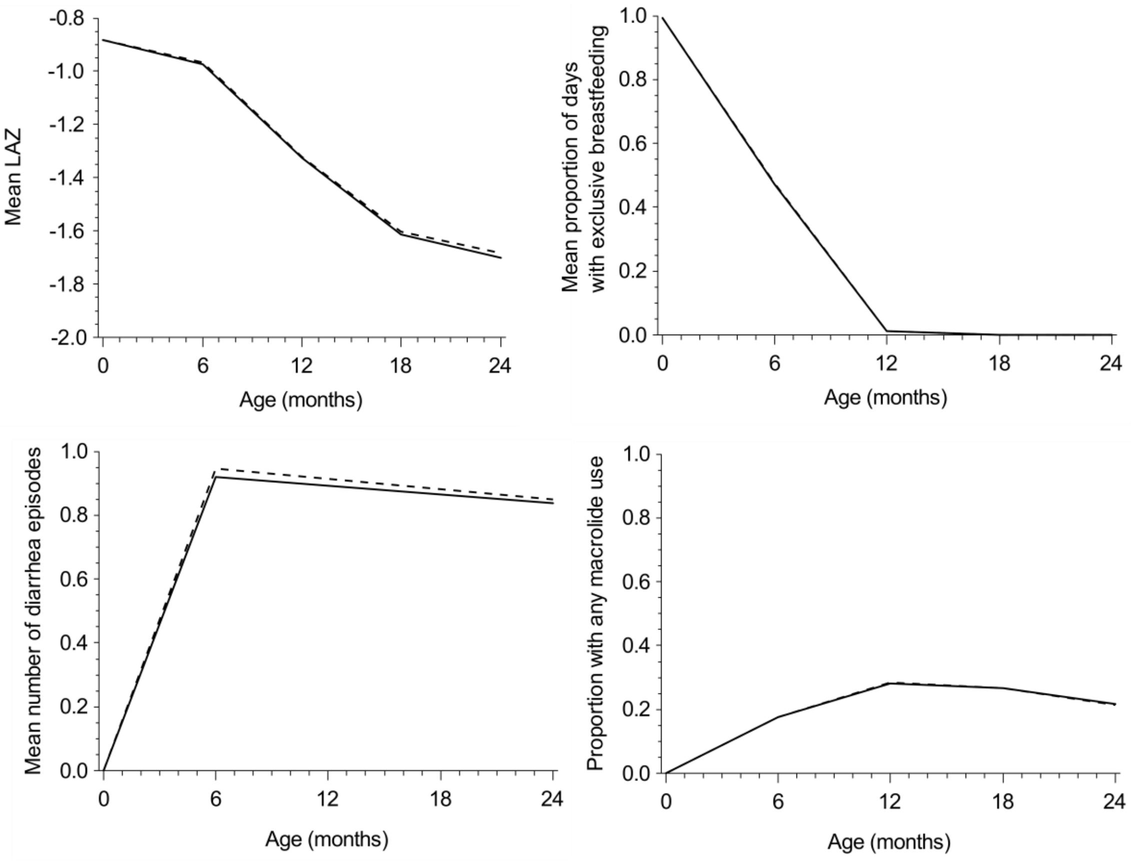

Figure S3. Validation of parametric g-formula models for time-varying covariates and LAZ outcomes. Average LAZ, proportion of days with exclusive breastfeeding, number of diarrhoea episodes, and macrolide use from birth to 2 years of age in the observed data (solid line) and natural course parametric g-formula scenario (dotted line) based on 1469 children in the MAL-ED cohort.

Enteropathogen prevalence and quantity

Table S2. Enteropathogen prevalence in non-diarrhoeal stools during the first 2 years of life by site among 1469 children in the MAL-ED cohort.

		Percent non-diarrhoeal stools positive N (%)							
	Site	All	Dhaka, Bangladesh	Vellore, India	Bhaktapur, Nepal	Fortaleza, Brazil	Loreto, Peru	Venda, South Africa	Haydom, Tanzania
Bacteria	*Shigella*	3237 (10·8)	564 (13·1)	592 (12·4)	290 (5·8)	139 (4·9)	574 (13·6)	321 (7·0)	757 (17·8)
	EAEC	15314 (51·0)	1877 (43·6)	3052 (63·9)	2503 (49·6)	857 (30·1)	2621 (62·2)	1654 (35·9)	2750 (65·0)
	Campylobacter	8398 (28·0)	1702 (39·4)	1177 (24·7)	1325 (26·3)	428 (15·0)	1044 (24·8)	525 (11·4)	2197 (51·8)
	tEPEC	3653 (12·2)	862 (20·0)	770 (16·2)	344 (6·8)	97 (3·4)	590 (14·0)	201 (4·4)	789 (18·6)
	ETEC	9056 (30·0)	2026 (46·8)	1471 (30·7)	1057 (20·9)	222 (7·7)	1349 (32·0)	578 (12·6)	2353 (54·8)
	aEPEC	8054 (26·8)	1083 (25·1)	1339 (28·1)	1535 (30·4)	742 (25·9)	1203 (28·6)	924 (20·2)	1228 (28·8)
Viruses	Norovirus	4338 (14·5)	836 (19·4)	730 (15·4)	628 (12·4)	161 (5·7)	806 (19·1)	477 (10·5)	700 (16·5)
	Astrovirus	2862 (9·6)	792 (18·3)	492 (10·4)	253 (5·0)	45 (1·6)	695 (16·6)	313 (6·8)	272 (6·4)
	Adenovirus 40/41	3791 (12·7)	779 (18·0)	787 (16·5)	296 (5·9)	126 (4·4)	969 (23·3)	491 (10·7)	343 (8·1)
	Sapovirus	3680 (12·3)	746 (17·3)	662 (13·9)	536 (10·6)	122 (4·3)	660 (15·7)	501 (10·9)	453 (10·7)
Protozoa	*Giardia*	8898 (29·7)	923 (21·4)	1804 (37·6)	1036 (20·5)	442 (15·5)	1760 (42·3)	1099 (23·6)	1834 (43·8)
	Cryptosporidium	2363 (7·9)	364 (8·4)	257 (5·4)	318 (6·3)	38 (1·3)	528 (12·5)	250 (5·5)	608 (14·3)
	*E. bieneusi**	1674 (5·6)	354 (8·2)	135 (2·8)	161 (3·2)	19 (0·7)	256 (6·1)	190 (4·2)	559 (13·2)

**E. bieneusi* is an intracellular parasitic fungus

Table S3. Enteropathogen quantity in positive non-diarrhoeal stools during the first 2 years of life by site among 1469 children in the MAL-ED cohort.

		Mean quantity in log (copy number) per gram of stool (standard deviation)							
	Site	All	Dhaka, Bangladesh	Vellore, India	Bhaktapur, Nepal	Fortaleza, Brazil	Loreto, Peru	Venda, South Africa	Haydom, Tanzania
Bacteria	*Shigella*	5·62 (1·29)	5·61 (1·19)	5·47 (1·20)	5·64 (1·26)	5·14 (1·24)	5·81 (1·43)	5·05 (0·94)	5·91 (1·37)
	EAEC	6·12 (1·52)	5·97 (1·56)	6·06 (1·46)	6·59 (1·46)	5·66 (1·37)	6·48 (1·65)	5·35 (1·09)	6·14 (1·52)
	Campylobacter	5·74 (1·33)	5·95 (1·36)	5·37 (1·21)	6·06 (1·41)	4·88 (0·85)	5·85 (1·37)	4·91 (1·14)	5·91 (1·21)
	tEPEC	5·83 (1·43)	5·79 (1·49)	5·71 (1·37)	6·28 (1·34)	5·38 (1·18)	5·88 (1·55)	5·37 (1·08)	5·92 (1·41)
	ETEC	5·88 (1·60)	5·68 (1·56)	5·65 (1·49)	6·47 (1·64)	5·47 (1·38)	5·89 (1·73)	5·27 (1·17)	6·12 (1·59)
	aEPEC	5·45 (1·24)	5·46 (1·26)	5·35 (1·19)	5·79 (1·23)	5·30 (1·13)	5·70 (1·37)	4·88 (0·91)	5·40 (1·25)
Viruses	Norovirus	5·25 (1·10)	5·29 (1·10)	5·18 (1·03)	5·27 (1·14)	5·01 (1·00)	5·41 (1·14)	5·05 (1·03)	5·29 (1·10)
	Astrovirus	5·37 (1·71)	5·25 (1·55)	5·17 (1·55)	6·17 (1·99)	5·64 (1·93)	5·22 (1·68)	5·15 (1·55)	5·89 (2·00)
	Adenovirus 40/41	5·35 (1·64)	5·28 (1·44)	5·17 (1·47)	6·10 (2·36)	5·55 (1·61)	5·22 (1·45)	5·00 (1·35)	6·08 (2·08)
	Sapovirus	5·55 (1·50)	5·63 (1·52)	5·37 (1·44)	5·69 (1·61)	5·53 (1·40)	5·50 (1·52)	5·38 (1·39)	5·83 (1·46)
Protozoa	*Giardia*	6·36 (1·77)	5·72 (1·66)	5·98 (1·63)	7·54 (1·74)	5·92 (1·32)	6·63 (1·78)	5·40 (1·45)	6·80 (1·67)
	Cryptosporidium	5·41 (1·34)	5·46 (1·47)	5·21 (1·13)	5·52 (1·37)	4·95 (1·08)	5·64 (1·47)	5·06 (1·15)	5·39 (1·25)
	*E. bieneusi**	5·63 (1·39)	5·87 (1·35)	5·68 (1·46)	5·67 (1·45)	5·45 (1·18)	5·62 (1·53)	5·37 (1·30)	5·54 (1·34)

**E. bieneusi* is an intracellular parasitic fungus

Table S4. Enteropathogen prevalence per child in 6 month intervals during the first 2 years of life among 1469 children in the MAL-ED cohort.

		Median percent non-diarrhoeal stools positive per child (10th, 90th percentiles)				
	Months	1-24	1-6	7-12	13-18	19-24
Bacteria	*Shigella*	0·09 (0–0·24)	0 (0–0)	0 (0–0·33)	0 (0–0·40)	0·17 (0–0·50)
	EAEC	0·50 (0·25–0·74)	0·4 (0–1)	0·6 (0·20–1)	0·5 (0·17–0·83)	0·5 (0–0·80)
	Campylobacter	0·24 (0·05–0·57)	0 (0–0·50)	0·33 (0–0·75)	0·25 (0–0·80)	0·2 (0–0·80)
	tEPEC	0·10 (0–0·25)	0 (0–0·25)	0·17 (0–0·40)	0 (0–0·33)	0 (0–0·33)
	ETEC	0·27 (0·06–0·57)	0·17 (0–0·5)	0·25 (0–0·75)	0·33 (0–0·75)	0·33 (0–0·75)
	aEPEC	0·26 (0·13–0·41)	0 (0–0·40)	0·33 (0–0·60)	0·33 (0–0·60)	0·33 (0–0·60)
Viruses	Norovirus	1·14 (0·04–0·26)	0 (0–0·33)	0·17 (0–0·50)	0·17 (0–0·40)	0 (0–0·33)
	Astrovirus	0·08 (0–0·22)	0 (0–0·33)	0 (0–0·33)	0 (0–0·33)	0 (0–0·25)
	Adenovirus 40/41	0·10 (0–0·27)	0 (0–0·33)	0 (0–0·40)	0 (0–0·33)	0 (0–0·33)
	Sapovirus	0·11 (0–0·23)	0 (0–0·20)	0·17 (0–0·40)	0·17 (0–0·40)	0 (0–0·33)
Protozoa	*Giardia*	0·25 (0–0·63)	0 (0–0·33)	0 (0–0·67)	0·2 (0–1)	0·6 (0–1)
	Cryptosporidium	0·05 (0–0·18)	0 (0–0)	0 (0–0·25)	0 (0–0·33)	0 (0–0·33)
	*E. bieneusi**	0 (0–0·16)	0 (0–0)	0 (0–0.33)	0 (0–0.33)	0 (0–0.33)

**E. bieneusi* is an intracellular parasitic fungus

Table S5. Enteropathogen quantity per child during the first 2 years of life among 1469 children in the MAL-ED cohort.

		Median quantity in log (copy number) per gram of stool (10th, 90th percentiles)				
	Months	1-24	1-6	7-12	13-18	19-24
Bacteria	*Shigella*	0·49 (0–1·39)	0 (0–0)	0 (0–1·85)	0 (0–2·27)	0·74 (0–3·02)
	EAEC	3·12 (1·37–4·69)	2·71 (0–6·28)	3·74 (0·98–6·39)	3·01 (0·82–5·44)	2·46 (0–4·71)
	Campylobacter	1·32 (0·27–3·30)	0 (0–2·78)	1·60 (0–4·35)	1·39 (0–4·52)	1·12 (0–4·21)
	tEPEC	0·62 (0–1·46)	0 (0–1·75)	0·72 (0–2·50)	0 (0–2·18)	0 (0–1·99)
	ETEC	1·63 (0·31–3·38)	0 (0–0)	1·56 (0–3·51)	1·52 (0–3·30)	1·39 (0–3·34)
	aEPEC	1·39 (0·63–2·30)	0 (0–1·32)	0·70 (0–2·23)	0·75 (0–2·17)	0 (0–1·89)
Viruses	Norovirus	0·71 (0·18–1·39)	0 (0–2·05)	0·92 (0–2·52)	0.75 (0–1·96)	0 (0–1·56)
	Astrovirus	0·43 (0–1·17)	0 (0–1·50)	0 (0–1·86)	0 (0–1·74)	0 (0–1·49)
	Adenovirus 40/41	0·56 (0–1·44)	0 (0–1·72)	0 (0–2·22)	0 (0–2·03)	0 (0–1·92)
	Sapovirus	0·62 (0–1·27)	0 (0–0)	0 (0–1·48)	0 (0–1·94)	0 (0–1·85)
Protozoa	*Giardia*	1·45 (0–4·28)	0 (0–1·40)	0 (0–3·95)	1·22 (0–6·77)	3·38 (0–7·40)
	Cryptosporidium	0·32 (0–0·99)	0 (0–1·84)	0·70 (0–2·57)	0·72 (0–2·41)	0·72 (0–2·16)
	*E. bieneusi**	0 (0–0·89)	0 (0–0)	0 (0–1.65)	0 (0–1.59)	0 (0–1.66)

**E. bieneusi* is an intracellular parasitic fungus

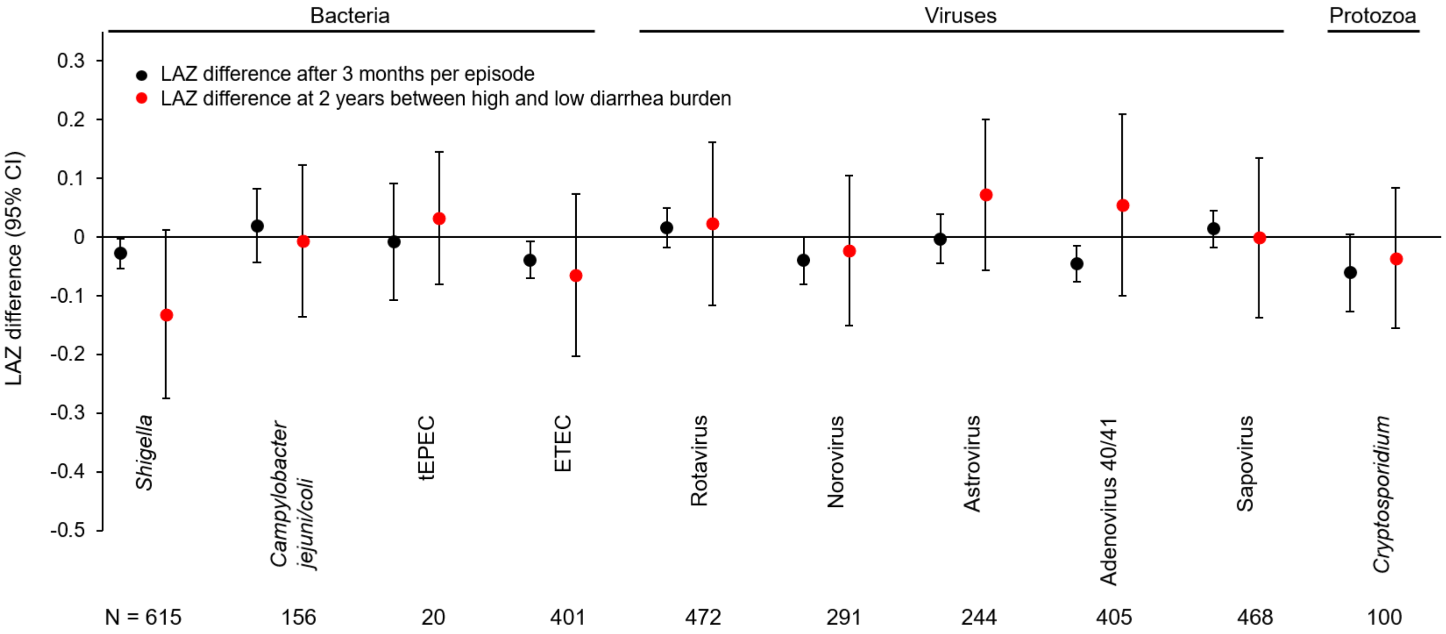

Figure S4. Associations of pathogen-attributable diarrhoea with length-for-age z-score.
Per-episode association of diarrhoea with length-for-age z-score (LAZ) 3 months after the episode (LAZ difference and 95% CI; black dots) and LAZ difference and 95% CI at 2 years of age between high (90th percentile) and low (10th percentile) pathogen-attributable diarrhoea burden (red dots). Analysis includes 37951 observed child-months among 1469 children in the MAL-ED cohort with molecular testing of stool samples. Per-episode estimates were adjusted for age, site, sex, SES, maternal height, LAZ at the beginning of the interval, exclusive breastfeeding, and non-attributable diarrhoea episodes in the same period. Two-year estimates were adjusted for site, sex, SES, maternal height, enrolment LAZ, exclusive breastfeeding in the first 6 months of life, number of antibiotic courses, and number of non-attributable diarrhoea episodes.

Site-specific effects

Table S6. Site-specific associations of pathogen-attributable diarrhoea episodes on LAZ 3 months following the episode adjusted for age, sex, SES, maternal height, and antibiotic treatment of the episode among 1469 children in the MAL-ED cohort.

	Diarrhoea aetiology			
Site	Any infectious aetiology	Bacterial*	Viral†	Parasitic‡
Dhaka, Bangladesh	-0·06 (-0·08 to -0·03)	-0·05 (-0·08 to -0·02)	-0·03 (-0·06–0·00)	0·01 (-0·10–0·11)
Vellore, India	-0·04 (-0·07 to -0·01)	-0·02 (-0·06–0·02)	-0·03 (-0·07–0·01)	-0·04 (-0·17–0·10)
Bhaktapur, Nepal	0·02 (-0·01–0·05)	0·00 (-0·05–0·05)	0·05 (0·01–0·08)	-0·14 (-0·26 to -0·02)
Fortaleza, Brazil	-0·10 (-0·23–0·02)	-0·03 (-0·19–0·12)	-0·10 (-0·27–0·07)	-0·27 (-0·60–0·05)
Loreto, Peru	-0·02 (-0·05–0·01)	-0·05 (-0·09 to -0·01)	0·01 (-0·02–0·05)	-0·10 (-0·19–0·00)
Venda, South Africa	0·05 (-0·06–0·17)	0·03 (-0·16–0·22)	0·05 (-0·10–0·19)	N/A§
Haydom, Tanzania	-0·13 (-0·22 to -0·04)	-0·04 (-0·18–0·09)	-0·15 (-0·28 to -0·01)	-0·15 (-0·41–0·11)

* Includes EAEC, aEPEC, tEPEC, ETEC, STEC, *Aeromonas*, *C. jejuni/coli*, *H. pylori*, *Plesiomonas*, *Salmonella*, *Shigella*, and *V. cholera*
† Includes adenovirus 40/41, astrovirus, norovirus GI/GII, rotavirus, sapovirus
‡ Includes *E. bieneusi*, *E. intestinalis*, *Cryptosporidium*, *Cyclospora*, *Isospora*, *E. histolytica*, *Giardia*, *Ancylostoma*, *Strongyloides*, and *Trichuris*
§ No parasite-attributable diarrhoea episodes were identified in Venda, South Africa

Table S7. Site-specific differences in length-for-age z-score at 2 years of age associated with the difference between high (site-specific 90th percentile) and low (site-specific 10th percentile) exposure to each of the 13 most prevalent enteropathogens in non-diarrhoeal stools among 1469 children in the MAL-ED cohort.

		All sites	Dhaka, Bangladesh	Vellore, India	Bhaktapur, Nepal
Bacteria	*Shigella*	-0·14 (-0·27 to -0·01)	-0·23 (-0·53–0·08)	0.00 (-0·28–0·28)	0·21 (-0·04–0·46)
	EAEC	-0·21 (-0·37 to -0·05)	-0·26 (-0·52–0·01)	0·11 (-0·16–0·39)	0·07 (-0·19–0·32)
	Campylobacter	-0·17 (-0·32 to -0·01)	-0·19 (-0·50–0·13)	-0·05 (-0·33–0·24)	-0·34 (-0·61 to -0·07)
	tEPEC	-0·11 (-0·27–0·05)	0·15 (-0·14–0·44)	-0·10 (-0·38–0·18)	-0·38 (-0·64 to -0·11)
	ETEC	0·10 (-0·11–0·31)	0·01 (-0·29–0·31)	-0·07 (-0·35–0·21)	0·04 (-0·23–0·30)
	aEPEC	0·08 (-0·05–0·20)	-0·03 (-0·31–0·25)	-0·18 (-0·47–0·11)	0·14 (-0·12–0·40)
Viruses	Norovirus	-0·06 (-0·19–0·07)	-0·25 (-0·53–0·02)	-0·26 (-0·53–0·01)	0·17 (-0·08–0·42)
	Astrovirus	-0·02 (-0·16–0·12)	-0·04 (-0·31–0·23)	-0·08 (-0·35–0·19)	0·02 (-0·23–0·28)
	Adenovirus 40/41	-0·03 (-0·17–0·11)	-0·08 (-0·35–0·19)	0·12 (-0·14–0·38)	0·14 (-0·10–0·38)
	Sapovirus	0·04 (-0·09–0·16)	-0·15 (-0·43–0·13)	0·18 (-0·09–0·44)	0·16 (-0·09–0·41)
Protozoa	*Giardia*	-0·17 (-0·30 to -0·05)	0·09 (-0·18–0·36)	-0·25 (-0·52–0·03)	-0·18 (-0·43–0·06)
	Cryptosporidium	0·02 (-0·11–0·15)	0·23 (-0·04–0·51)	-0·04 (-0·30–0·22)	-0·18 (-0·45–0·09)
	*E· bieneusi**	-0·14 (-0·27 to -0·01)	0·09 (-0·19–0·38)	-0·05 (-0·32–0·21)	-0·15 (-0·39–0·10)

		Fortaleza, Brazil	Loreto, Peru	Venda, South Africa	Haydom, Tanzania
Bacteria	*Shigella*	-0·03 (-0·48–0·42)	-0·23 (-0·51–0·04)	-0·23 (-0·55–0·08)	-0·36 (-0·67 to -0·04)
	EAEC	-0·28 (-0·72–0·16)	-0·04 (-0·31–0·23)	-0·34 (-0·67–0.00)	-0·40 (-0·72 to -0·07)
	Campylobacter	-0·01 (-0·4–0·38)	0·08 (-0·19–0·34)	-0·30 (-0·61–0·01)	-0·06 (-0·37–0·24)
	tEPEC	0.00 (-0·39–0·39)	0·09 (-0·19–0·37)	-0·11 (-0·42–0·20)	-0·03 (-0·36–0·31)
	ETEC	0·14 (-0·28–0·56)	-0·17 (-0·45–0·11)	0·11 (-0·22–0·44)	0·38 (0·06–0·71)
	aEPEC	-0·17 (-0·59–0·26)	0·31 (0·03–0·60)	0·13 (-0·18–0·45)	0·31 (-0·01–0·63)
Viruses	Norovirus	0·06 (-0·35, 0·47)	-0·18 (-0·46–0·10)	0·03 (-0·29–0·35)	0·18 (-0·12–0·47)
	Astrovirus	-0·55 (-0·94 to -0·16)	-0·13 (-0·39–0·12)	0·16 (-0·14–0·47)	0·20 (-0·09–0·48)
	Adenovirus 40/41	-0·01 (-0·43–0·41)	-0·14 (-0·41–0·13)	-0·19 (-0·49–0·11)	-0·04 (-0·32–0·25)
	Sapovirus	0·33 (-0·09–0·75)	0·16 (-0·10–0·43)	0·05 (-0·26–0·36)	-0·19 (-0·48–0·10)
Protozoa	*Giardia*	0·16 (-0·28–0·61)	-0·33 (-0·60 to -0·05)	-0·35 (-0·66 to -0·04)	-0·10 (-0·40–0·21)
	Cryptosporidium	-0·03 (-0·43–0·37)	0·03 (-0·23–0·29)	0·38 (0·06–0·69)	-0·07 (-0·37–0·23)
	*E· bieneusi**	-0·06 (-0·46–0·34)	-0·19 (-0·46–0·07)	-0·24 (-0·55–0·07)	-0·24 (-0·54–0·05)

**E. bieneusi* is an intracellular parasitic fungus

Height attainment model sensitivity analyses

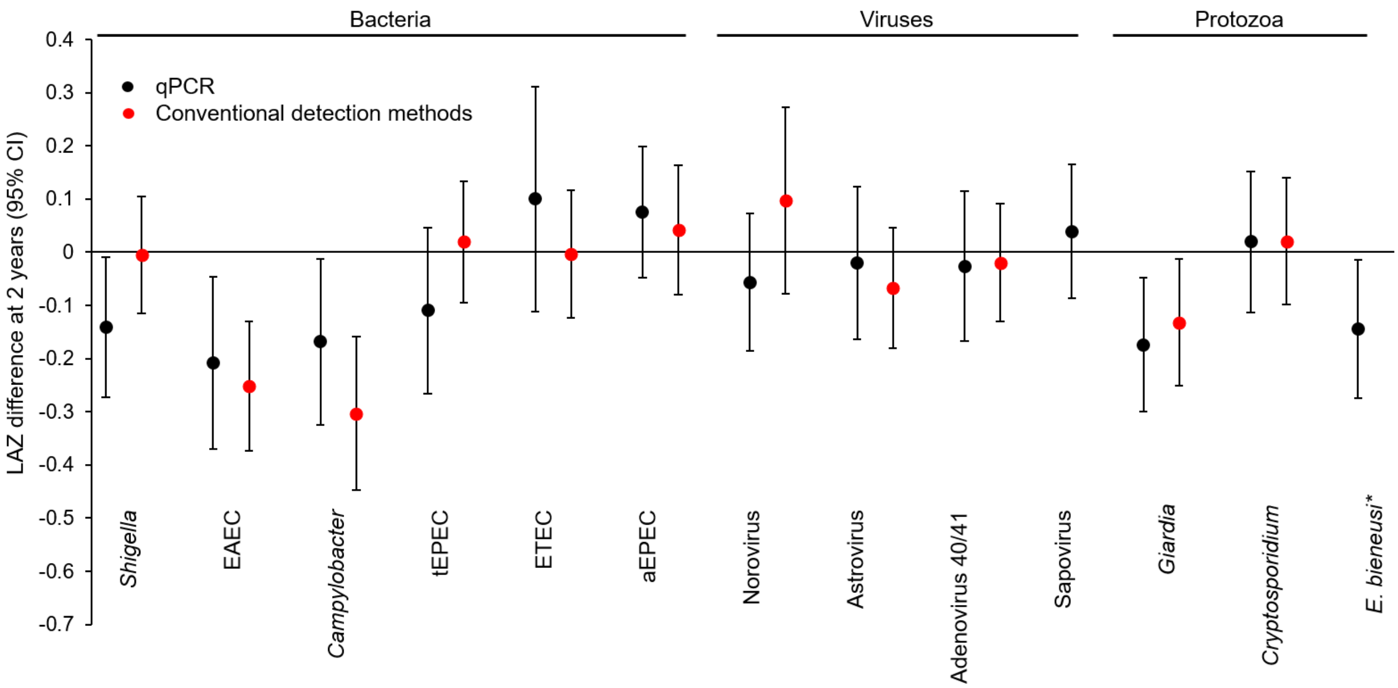

Figure S5. Height attainment model: qPCR vs. conventional methods.
Difference and 95% confidence interval in length-for-age z-score (LAZ) at 2 years of age associated with the difference between high (90th percentile) and low (10th percentile) pathogen prevalence for each of the 13 most prevalent enteropathogens using quantitative PCR detection methods (black) and conventional detection methods[5] (Table S1; red) in non-diarrhoeal stools among 1469 children in the MAL-ED cohort. Estimates are adjusted for site, enrolment LAZ, sex, SES, exclusive breastfeeding in the first 6 months of life, and maternal height.

Note: Sapovirus and *E. bieneusi* were not assayed using conventional methods and norovirus was only tested using conventional methods in random 10% of children. Conventional methods were conducted in non-diarrhoeal stools monthly in the first year of life and only quarterly in the second year, while qPCR was conducted in non-diarrhoeal stools monthly in both years.

**E. bieneusi* is an intracellular parasitic fungus

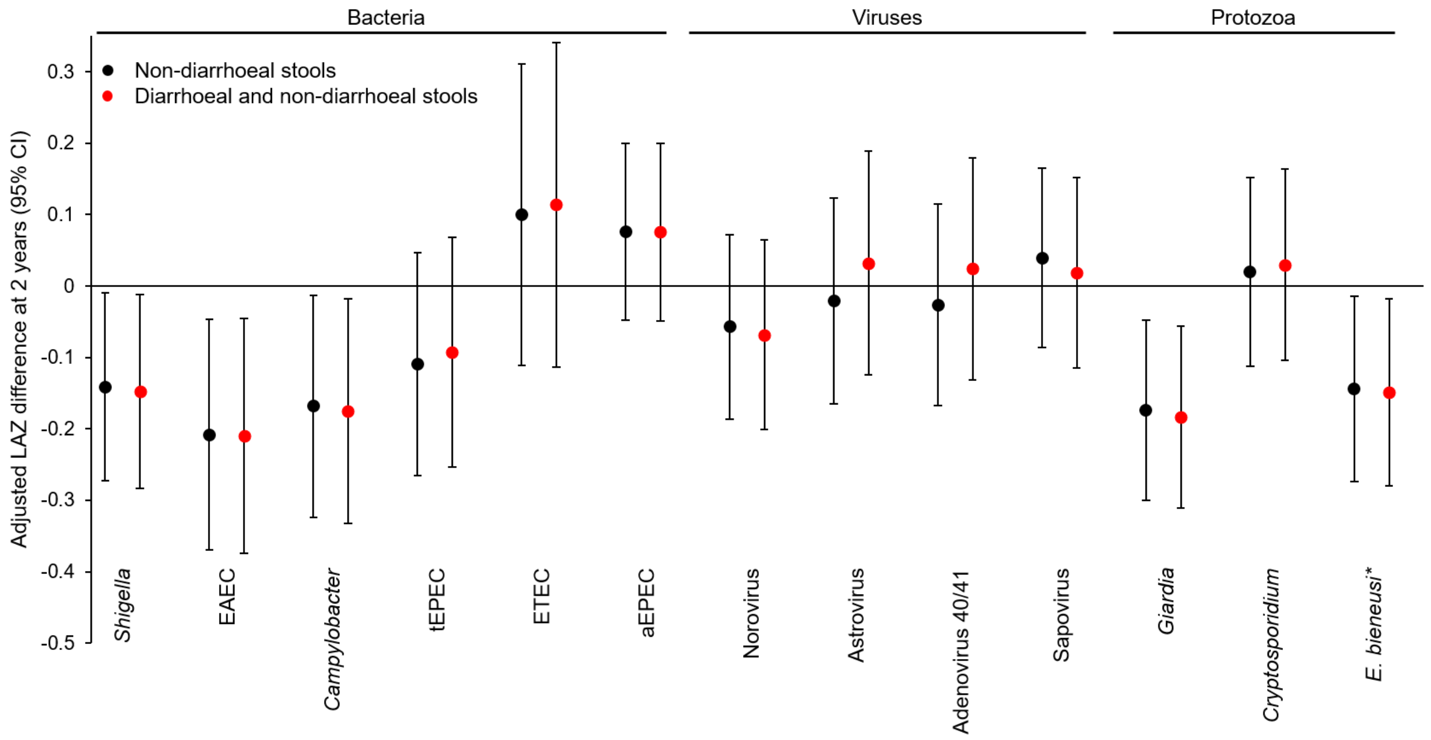

Figure S6. Height attainment model: non-diarrhoeal stools and diarrhoeal stools.
Difference and 95% confidence interval in LAZ at 2 years of age associated with the difference between high (90th percentile) and low (10th percentile) pathogen prevalence for each of the 13 most prevalent enteropathogens in non-diarrhoeal stools (black) and all stools (diarrhoeal and non-diarrhoeal; red) among 1469 children in the MAL-ED cohort. Estimates are adjusted for site, enrolment WAZ and LAZ, sex, SES, exclusive breastfeeding in the first 6 months of life, and maternal height.

**E. bieneusi* is an intracellular parasitic fungus

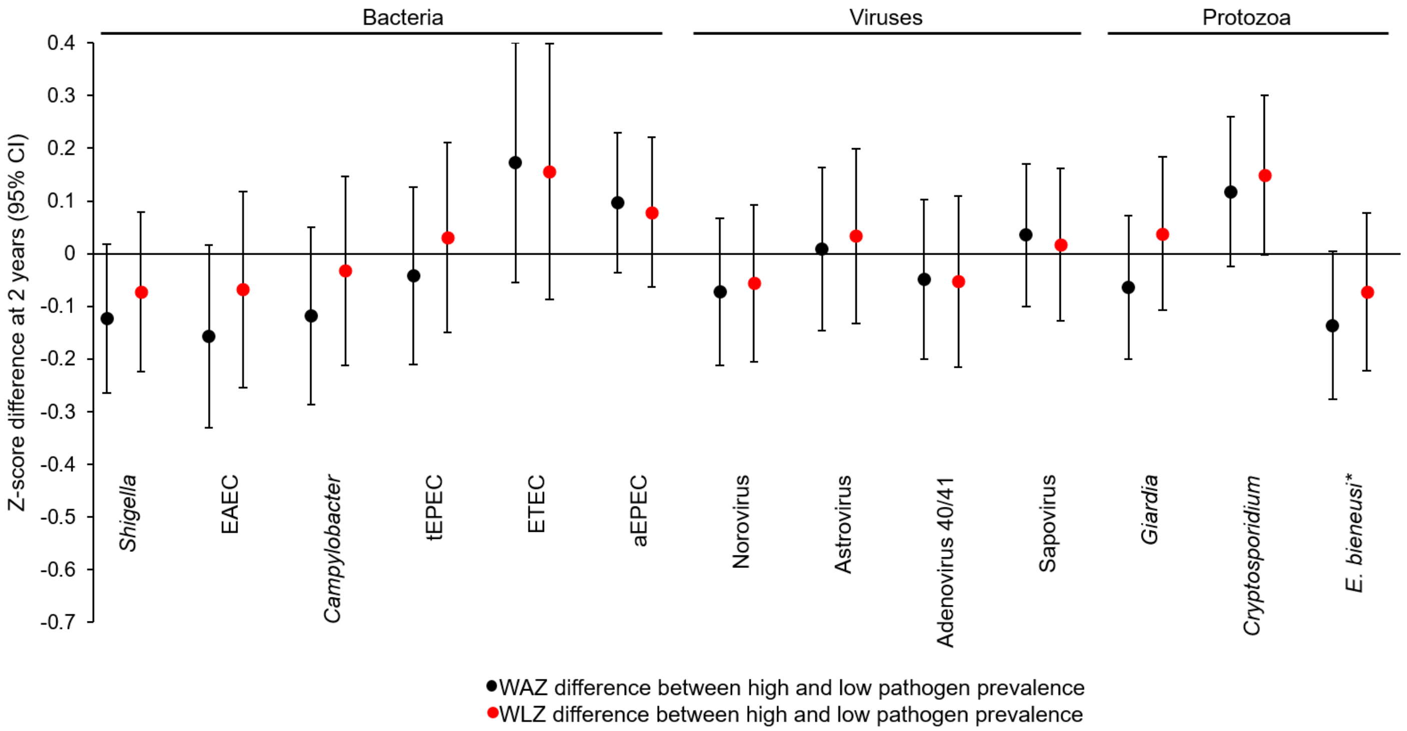

Figure S7. Height attainment model: weight-for-age (WAZ) and weight-for-length (WLZ) z-scores. Difference and 95% confidence interval in WAZ and WLZ at 2 years of age associated with the difference between high (90th percentile) and low (10th percentile) pathogen prevalence for each of the 13 most prevalent enteropathogens in non-diarrhoeal stools among 1469 children in the MAL-ED cohort. Estimates are adjusted for site, enrolment WAZ and LAZ, sex, SES, exclusive breastfeeding in the first 6 months of life, and maternal height.

**E. bieneusi* is an intracellular parasitic fungus

Longitudinal model sensitivity analyses

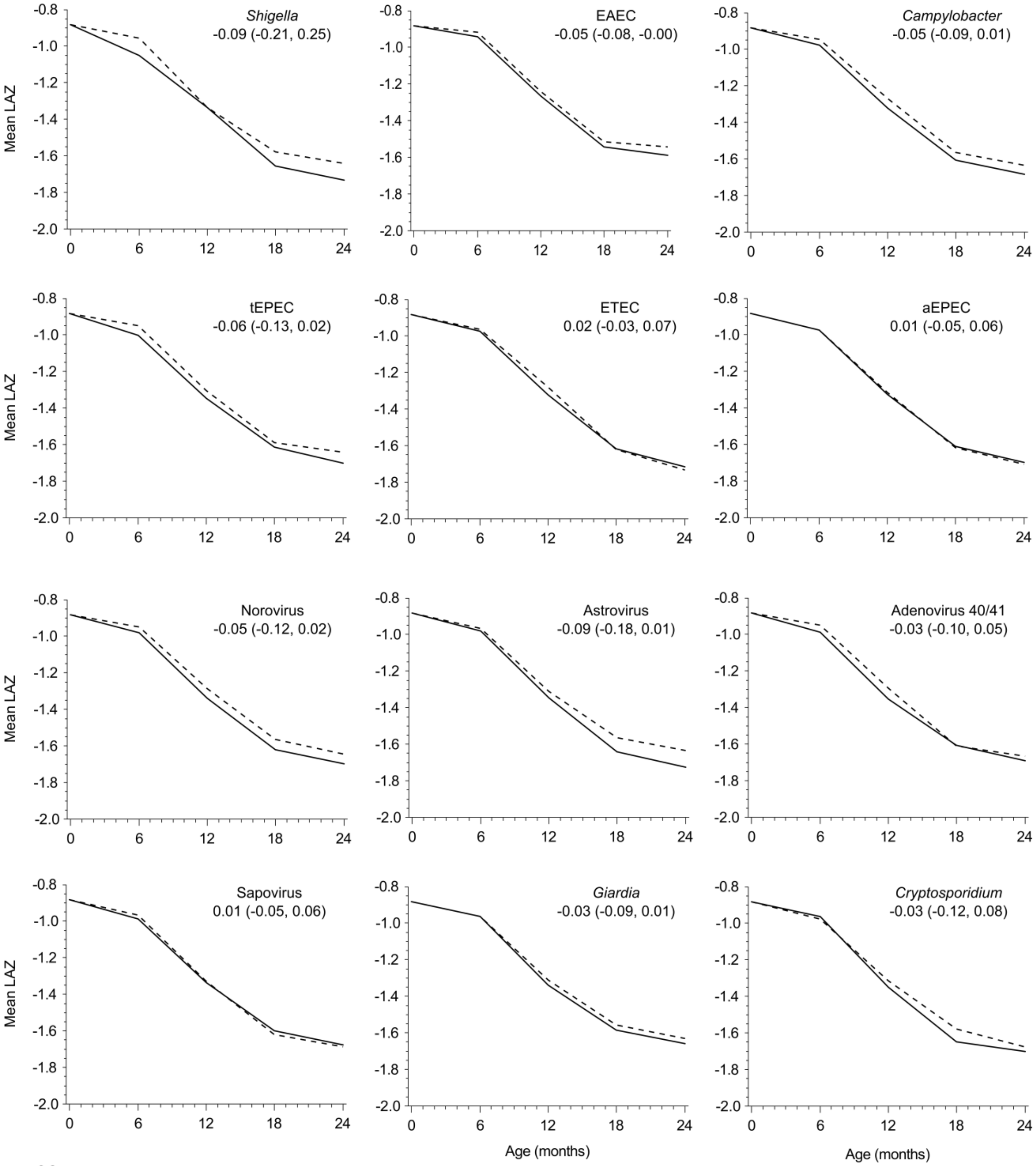

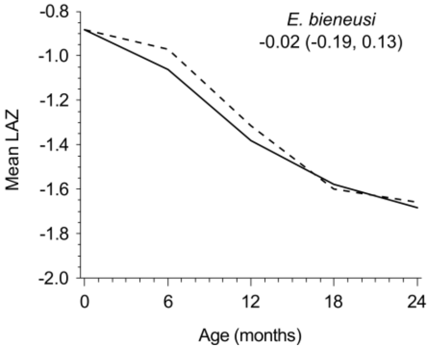

Figure S8. Longitudinal g-formula: effects scaled per one log increase in pathogen quantity.
Mean length-for-age z-score (LAZ) from birth to 2 years of age comparing parametric g-formula scenarios that represent a one log increase in enteropathogen quantity per gram of stool (solid line compared to dotted line), for the 13 most prevalent enteropathogens in non-diarrhoeal stools. The LAZ difference and 95% CI at 2 years comparing the two scenarios is overlaid on each graph.

References

1 Liu J, Gratz J, Amour C, *et al.* Optimization of Quantitative PCR Methods for Enteropathogen Detection. *PloS One* 2016; **11**: e0158199.

2 Liu J, Platts-Mills JA, Juma J, *et al.* Use of quantitative molecular diagnostic methods to identify causes of diarrhoea in children: a reanalysis of the GEMS case-control study. *Lancet Lond Engl* 2016; **388**: 1291–301.

3 Liu J, Gratz J, Amour C, *et al.* A Laboratory-Developed TaqMan Array Card for Simultaneous Detection of 19 Enteropathogens. *J Clin Microbiol* 2013; **51**: 472–80.

4 Greenland S, Pearl J, Robins JM. Causal diagrams for epidemiologic research. *Epidemiol Camb Mass* 1999; **10**: 37–48.

5 Houpt E, Gratz J, Kosek M, *et al.* Microbiologic methods utilized in the MAL-ED cohort study. *Clin Infect Dis Off Publ Infect Dis Soc Am* 2014; **59 Suppl 4**: S225–32.
